# Health-related quality of life in paediatric patients with Type 1 diabetes mellitus using insulin infusion systems. A systematic review and meta-analysis

**DOI:** 10.1371/journal.pone.0217655

**Published:** 2019-06-25

**Authors:** Bastian Rosner, Andres Roman-Urrestarazu

**Affiliations:** 1 Institute of Public Health, University of Cambridge, Forvie Site, Robinson Way, Cambridge, United Kingdom; 2 Faculty of Health, Medicine and Life Sciences, Department of International Health, University of Maastricht, Maastricht, Netherlands; University of Mississippi Medical Center, UNITED STATES

## Abstract

**Background:**

In 2017, more than 1.1 million children were living with type 1 diabetes mellitus (T1DM) globally. The goal in paediatric diabetes therapy is reaching optimal glycaemic control as early as possible in order to avoid complications and early mortality without compromising the quality of life (QoL) of children. Several different insulin regimens are available for T1DM patients to reach this goal.

**Aims:**

This review set out to analyse whether continuous subcutaneous insulin infusion (CSII) regimens are superior to multiple daily injection (MDI) therapy in T1DM youth regarding QoL. Additionally, it assessed glycaemic control and adverse events as secondary outcomes and discussed potential future public health implications and justifications for using CSII as a first-line therapy in diabetic youth.

**Methods:**

A systematic review and random effects meta-analysis was performed on studies investigating the association between QoL and diabetes treatment regimen. Differences in adverse event rates between groups were analysed using a Mann-Whitney U test. Lastly, differences in glycaemic control were assessed using a random effects meta-analysis.

**Results:**

QoL and glycaemic control was significantly better in CSII subjects at baseline and follow-up. No significant differences in adverse events were found between study groups. No significant changes over time could be shown for either QoL or glycaemic control.

**Conclusion:**

CSII proved to provide similar or slightly better outcomes in all analysed fields. This is consistent with previous research. However, to make credible recommendations, better-designed studies are needed to investigate the impact of CSII in children.

## Introduction

Diabetes mellitus (DM) is one of the top ten causes of global mortality, having killed 1.6 million people in 2016 alone.[1–3] DM describes a cluster of metabolic diseases, rather than a single illness, that are characterised by chronic hyperglycaemia.[4] The American Diabetes Association (ADA) classifies DM into four general categories with the most common ones being type 2 diabetes mellitus (T2DM) and type 1 diabetes mellitus (T1DM) following in second place.[4, 5] It is estimated that more than 96,000 children under the age of fifteen are diagnosed with T1DM annually, whilst there are 1.1 million children and adolescents below 20 years living with T1DM globally.[6] There are considerable regional differences in the prevalence of T1DM with more than one quarter (28.4%) of paediatric patients living in Europe and more than one fifth (21.5%) living in North America and the Caribbean.[6] The highest incidence of T1DM can be seen in the United States (US), India and Brazil.[6] Complications in T1DM are relatively frequent and can be divided into acute (e.g. diabetic ketoacidosis, infection) and chronic (macro- and microangiopathy). In addition to being a global health problem due to its multiple short and long-term complications, diabetes and related conditions account for an enormous economic burden throughout the world.[7] This burden is expected to continue growing with a projected expenditure of 776 billion US-Dollar by 2045 for adult patients only.[6] T1DM’s physiopathology is primarily due to β-cell destruction and absolute insulin deficiency.[4] Thus, the therapeutic goal for T1DM patients is defined as reaching optimal glycaemic control as early as possible to avoid acute and chronic complications without compromising the quality of life (QoL) and wellbeing of children, their parents or caregivers.[8] The only way to reach this goal for patients with T1DM is—additionally to behavioural interventions—the uninterrupted supply of insulin.[6] Insulin regimens available for T1DM patients can be divided into three groups: multiple daily injection basal-bolus insulin regimens (MDI), mixed (biphasic) regimens and continuous subcutaneous insulin infusion regimens (CSII, insulin pump). Despite MDI still being the first-line therapy in many regions around the world[9–11] CSII is gaining popularity among paediatric patients.[12] This can be explained to some extent by slightly better metabolic control and less acute complications through CSII[12–14] but might also be influenced by other factors not yet fully understood.[8] With CSII being much more expensive than MDI–treatment cost would increase by 50% if all T1DM patients used CSII[15]–methodologically well-conducted studies are needed to prove its superiority over MDI and to justify it as a first-line choice.

So far, reviews investigating insulin pump therapy showed mixed results regarding the health-related quality of life (HRQOL) in paediatric diabetes patients.[13, 16, 17] According to recent publications,[15, 18–20] there is still a lack of adequately powered studies to underpin the advantages of CSII regarding QoL improvement for children diagnosed with DM and to potentially balance the higher treatment cost attached to it.[8] Thus, insulin pumps are–forty years after they were first introduced to the market–still not part of first-line recommendations in most countries around the world. The aim of this systematic review and meta-analysis is to analyse available evidence on whether CSII is superior to MDI therapy in T1DM youth regarding HRQOL. In addition, this work will assess glycaemic control and adverse events as secondary outcomes, since a close relationship between HRQOL and glycaemic control has been previously described. A thorough understanding of the links between both outcomes could have important implications for the adoption of CSII in paediatric diabetes care.[18] Finally, the paper will discuss the potential future public health significance and whether there is justification for using CSII as a first-line therapy in children and adolescents.

## Methods

T1DM is the most common type of diabetes in children.[6] Although T2DM is becoming more common in children and adolescents in some regions around the world, sufficient and reliable data on T2DM in childhood is sparse which makes an analysis of its global health impact difficult. Whereas T1DM can only be treated by insulin injections, there are multiple options for T2DM. Thus, this work will focus on paediatric patients with T1DM only. The definition of childhood provided by the World Health Organization (WHO) was used to set an age threshold for study inclusion criteria. According to the WHO, an adolescent is a person “10 to 19 years inclusive” and a child “is a person 19 years or younger”.[21] Therefore, studies including participants older than 19 years were excluded.

### Eligibility criteria

Studies were selected for inclusion according to the criteria stated (Table 1). Eligibility criteria were formulated before the primary literature search.

**Table 1:** Inclusion and exclusion criteria for study selection.

Inclusion criteria	Exclusion criteria
**1. Studies comparing quality of life between CSII regimens and MDI** **2. Studies solely focused on children and adolescents (WHO definition) with T1DM** **3. Primary data analysis** **4. Full-text accessible at University of Cambridge or University of Groningen** **5. Language: English** **6. Geographic region: Worldwide**	1. Studies comparing quality of life between CSII regimens and control groups other than MDI (e.g. healthy controls, other pump regimens) 2. Studies solely referring to closed-loop systems or sensor-augmented pump therapy 3. Studies focused on type 2 diabetes mellitus (T2DM) specifically 4. Studies not primarily assessing quality of life 5. No restriction to children and/or adolescents (WHO definition) 6. Full-text not accessible at University of Cambridge or University of Groningen 7. Languages other than English 8. Review, meta-analysis

## Literature search strategy

**Fig 1:**
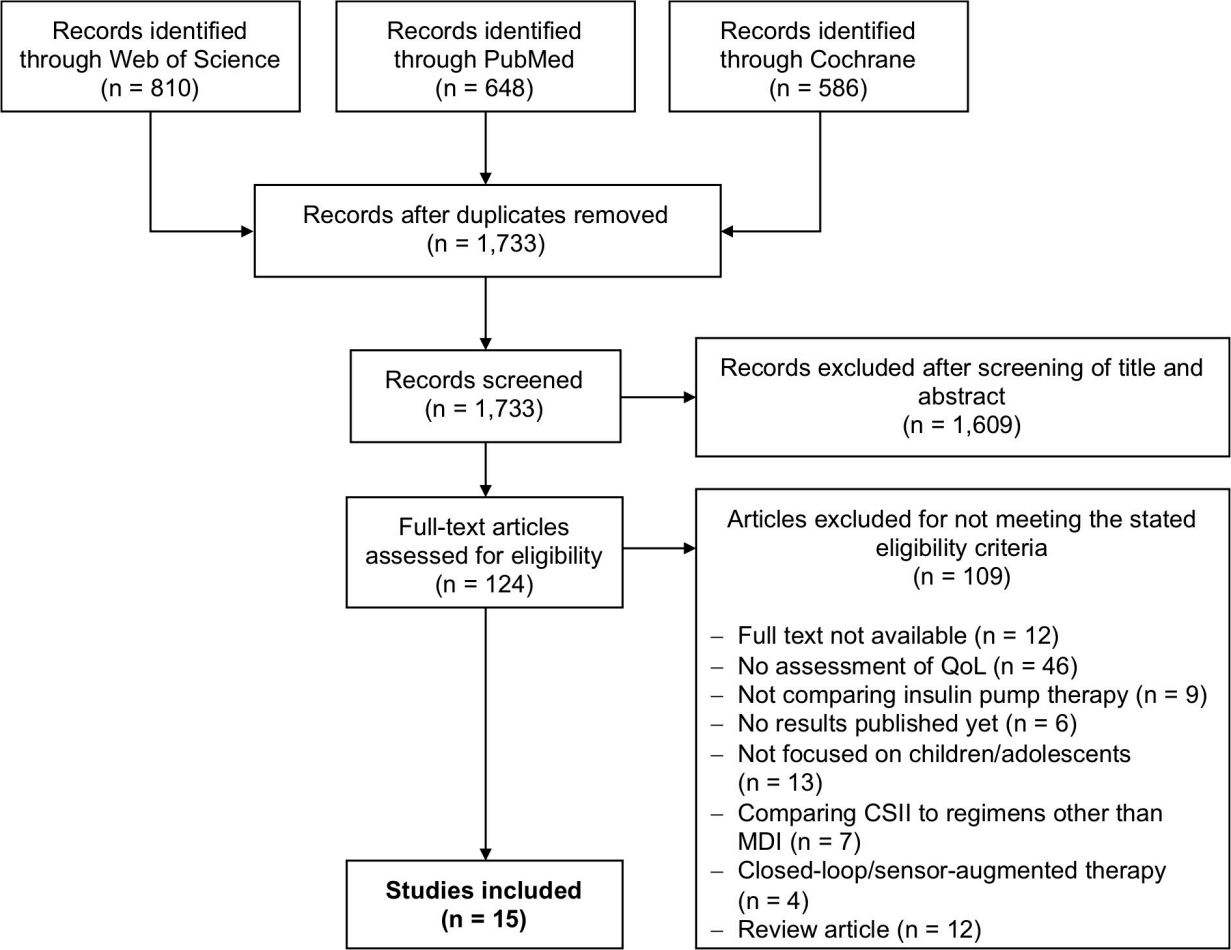
Literature search strategy.

A systematic literature search was performed on 12 December 2018 using PubMed, Web of Science and the Cochrane Library as primary data sources (Table 2 and Fig 1). Studies were selected upon meeting the eligibility criteria stated in Table 1. Additionally, Google.com was searched for grey literature and supplementary data sources. Also, reference lists of included studies and past reviews were screened for more relevant articles. Two levels of screening by two independent researchers (B. Rosner and A. Roman-Urrestarazu) were used on all citations. Our electronic search yielded 1,733 articles (Fig 1). We reviewed the titles and abstracts and eliminated any articles that clearly fell outside our inclusion/exclusion criteria. If there was any doubt, the article was retained for the next level of scrutiny. This process yielded 124 articles. Two authors examined each article’s title and abstract more closely and, if needed, examined the full text of each article and made independent judgments as to whether the article met inclusion and exclusion criteria. Disagreements were resolved by face-to-face discussion, leading to a consensus judgement. Fifteen articles met our inclusion and exclusion criteria.

**Table 2:** Search terms included in database search.

Database	Search Strategy
**PubMed**	1. quality of life **OR** QoL **OR** quality of life [MeSH] **AND** 2. diabetes **OR** diabetes mellitus type 1 **OR** insulin‐dependent diabetes **OR** diabetes mellitus, type 1 [MeSH] **AND** 3. insulin **OR** continuous subcutaneous infusion **OR** continuous subcutaneous injection **OR** CSII **OR** pump therapy **OR** insulin infusion systems [MeSH] **AND** 4. children **OR** child* **OR** newborn **OR** infant* **OR** teenagers **OR** teenag* **OR** adolescent* **OR** child [MeSH] **OR** adolescent [MeSH] **OR** infant [MeSH] **OR** child, preschool [MeSH] **OR** newborn [MeSH] *Filters*: *Humans; English*
**Web of Science**	1. quality of life **OR** QoL **AND** 2. diabetes **OR** diabetes mellitus type 1 **OR** insulin‐dependent diabetes **AND** 3. insulin **OR** continuous subcutaneous infusion **OR** continuous subcutaneous injection **OR** CSII **OR** pump therapy **AND** 4. children **OR** child* **OR** newborn **OR** infant* **OR** teenagers **OR** teenag* **OR** adolescent* Filters: *English; all years; all fields*
**Cochrane**	1. quality of life **OR** QoL **OR** quality of life [MeSH] **AND** 2. diabetes **OR** diabetes mellitus type 1 **OR** insulin‐dependent diabetes **OR** diabetes mellitus, type 1 [MeSH] **AND** 3. insulin **OR** continuous subcutaneous infusion **OR** continuous subcutaneous injection **OR** CSII **OR** pump therapy **OR** insulin infusion systems [MeSH] **AND** 4. children **OR** child* **OR** newborn **OR** infant* **OR** teenagers **OR** teenag* **OR** adolescent* **OR** child [MeSH] **OR** adolescent [MeSH] **OR** infant [MeSH] **OR** infant, newborn [MeSH] *No filters used*.

### Quality assessment of included studies and literature bias analysis

The Effective Public Health Practice Project Tool (EPHPP) was used to assess and compare the quality of included studies.[22, 23] The EPHPP allows for the evaluation of internal (study design, confounding, blinding, withdrawals and dropouts, intervention integrity, analyses) as well as external (data collection methods, selection bias) validity of studies.[24] The quality assessment results are summarised in the S1 Table.

We used ratio measures of intervention effect (Odds ratios) and plotted on a logarithmic scale to evaluate possible literature bias in Funnel Plots, using Egger test as well. The aim of this was to ensure that effects of the same magnitude but opposite directions were equidistant from 1. For outcomes measured on a continuous (numerical) scale (e.g. blood pressure, depression score) intervention effects were measured as standardised mean differences. For mean differences, the standard error was approximately proportional to the inverse of the square root of the number of participants.

### Data collection and quantitative analysis

The data extracted from each study comprised lead author, sample characteristics, study setting, study design, follow-up details, information on exposure and outcome measurements as well as on confounders. Means were used as the main measures of association across studies. If more than one effect size was reported for the same relationship, the maximally adjusted model was chosen. A comparison between baseline and follow-up data was not always possible due to differences in study design between papers. To analyse the association between HRQOL and insulin treatment regimen standardised mean differences (SMD) were calculated for each study (see below) at two time points (baseline and follow-up) using the reported mean HRQOL scores for each group (CSII and MDI). This approach is endorsed by the Cochrane Collaboration when studies included in a meta-analysis assess the same outcome but measure it in a variety of ways (e.g. use different assessment tools).[25] SMD were entered into the random effects meta-analysis as primary effect measures.

$$\mathrm{SMD:}\frac{\mathrm{Difference}\;\mathrm{in}\;\mathrm{mean}\;\mathrm{outcome}\;\mathrm{between}\;\mathrm{groups}}{\mathrm{Standard}\;\mathrm{deviation}\;\mathrm{of}\;\mathrm{outcome}\;\mathrm{among}\;\mathrm{participants}}$$

Due to heterogeneity in used QoL assessment tools a Mann-Whitney U test was carried out to look for potential associations between adverse event rates per patient year and the assigned treatment groups, the hypothesis being that lower incidence of adverse events would create a higher QoL.[26, 27] Additionally, HbA1c (glycated haemoglobin in %) was assessed as a secondary outcome to evaluate the effectiveness of the different treatment regimens (glycaemic control) and to account for it as a potential confounder throughout studies. Mean differences (MD) between treatment groups at baseline and follow-up were retrieved from each paper and entered into a random effects meta-analysis to approximate an overall pooled effect size for each point in time. If HbA1c measures were not reported in per cent of total haemoglobin but in mmol/mol the following formula was used for conversion:[28]

HbA1c(%)=[0.09148*HbA1c(mmol/mol)]+2.152

Random effects models were chosen for all meta-analyses because of anticipated between-study variance. Heterogeneity was judged by using the Cochran Q test and the I^2^ statistic which gives the percentage of between-study variation attributable to heterogeneity.[29] If not stated otherwise the significance level was set to 0.05. All statistical analyses were performed using STATA IC Version 15.1.[30]

## Results

Fifteen eligible studies could be identified and were included in the analysis of this paper with an agreement percentage between raters of 89.5% and Cohen’s Kappa: 0.604 (Table 3). No additional data was included through screening of references and grey literature. The overall quality of studies was poor (S1 Table). Eligible papers were published between 2003 and 2018, sample sizes ranged from 16 to 700 and all studies included male and female patients in their analyses. Study centres were based in the US[31–34], Germany[8, 35], Denmark[36, 37], Italy[38], the Netherlands[39], Hungary[40], England and Wales[15], and Israel[41–43] (with one of the Israeli studies additionally including patients from a study site in Slovenia[41]). Research methodology varied substantially, particularly because of different study designs used. Six studies were randomised controlled trials (RCT)[8, 15, 33, 39, 42, 43] (of which two applied a crossover design[42, 43]), another six were cross-sectional studies (CSS)[31, 32, 34, 36, 38, 40] and one each were a clinical trial (CT)[37], a crossover CT[41] and a prospective observational study[35]. The age of study participants ranged from 0.6 to 19 years (Table 3). Three of the studies[8, 15, 35] stated results for different age groups with Mueller-Godeffroy et al.[8, 35] and Blair et al.[15] reporting for patient cohorts younger than 8 years, 8–11 years, 12–16 years and cohorts younger than 5 years, 5–11 years, 12–15 years respectively. Inclusion criteria typically consisted of all patients being diagnosed with T1DM, being currently treated with MDI (RCT) or being either treated with CSII or MDI (CSS) before the study. Only one paper included newly diagnosed T1DM patients with no prior treatment.[15] Given consent by patients and their parents as well as the absence of major comorbidities were additional eligibility criteria. All studies reported HbA1c and QoL as outcome measures, nine studies[15, 33, 35, 37–39, 41–43] stated the number of occurred adverse events within each study group (Table 4). Other reported secondary outcomes—which are not the focus of this review—were cardiorespiratory fitness, parenting stress, treatment satisfaction, insulin dose, BMI, hypoglycaemia fear and cost-effectiveness.

**Table 3:** Study characteristics for selected studies.

Lead author	Journal	Year	Study period	Total # of patients	Age range (years)	Location	Study type	Follow-up period (months)	Outcomes assessed*	QoL assessment tool	Adjustments
**Mueller-Godeffroy [8]**	Pediatric Diabetes	2018	2011–2014	211	6–16	Germany	RCT**	6	H, Q	KINDL-DM	Baseline, age, centre as a random factor
**Blair [15]**	Health Technology Assessment	2018	2011–2015	293	0.6–15	England/Wales	RCT	12	G, H, K, Q	PedsQL	Age, centre as a random factor
**Cherubini [38]**	Acta Diabetologica	2014	2008–2009	577	10–17	Italy	CSS***	N/A°	H, Q	IDSRQ	Age, gender, number of weekly hours spent in physical activity, basal insulin dose, self-administration of insulin, number of visits to the investigation centre
**Birkebaek [36]**	Diabetes Research and Clinical Practice	2014	2009	700	8–17	Denmark	CSS	N/A	H, Q	PedsQL	Gender, age, diabetes duration and HbA1c
**Rendell [31]**	Journal of Diabetes Science and Technology	2013	Not given	53	9–17	USA	CSS	N/A	H, Q	Community Assessment Instrument Pre-Test 17 & WHOQOL-BREF	Not given
**Lukacs [40]**	International Journal of Technology Assessment in Health Care	2013	Not given	239	8–18	Hungary	CSS	N/A	H, Q	PedsQL	VO_2_max value (maximum rate of oxygen consumption), insulin regimen, age, gender, diabetes duration, HbA1c, insulin dosage, BMI
**Wu [32]**	Diabetes Research and Clinical Practice	2010	Not given	62	12–17	USA	CSS	N/A	H, Q	DQOL-Y	Not given
**Mueller-Godeffroy [35]**	Diabetic Medicine	2009	2005–2006	117	4–16	Germany	Prospective observational study	6	G, H, Q	KINDL-DM	Not given
**Nuboer [39]**	Pediatric Diabetes	2008	Not given	38	4–16	Netherlands	Parallel RCT	3.5	G, H, K, Q	PedsQL	Baseline
**Johannesen [37]**	Pediatric Diabetes	2008	Not given	56	13–19	Denmark	CT****	12	G, H, K, Q	Not specified (“validated QoL questionnaire”)	Matching according to HbA1c, age, diabetes duration and gender.
**Wilson [33]**	Diabetes Care	2005	2001–2003	19	1–6	USA	RCT	12	G, H, K, Q	DQOL for toddlers	Not given
**O'Neil [34]**	Journal of the American Dietetic Association	2005	2003	103	9–17	USA	CSS	N/A	H, Q	DQOL-Y	Age, gender, diabetes diagnosis age, treatment regimen, HbA1c level, body mass index (BMI)
**Shehadeh [41]**	Israel Medical Association Journal	2004	Not given	15	1–5	Israel/Slovenia	Crossover CT	12	G, H, K, Q	DQOL, modified	Not given
**Weintrob [42]**	Pediatrics	2003	Not given	23	9–13	Israel	Crossover RCT	3.5	G, H, K, Q	DQOL-Y	Gender
**Cohen [43]**	Journal of Pediatric Endocrinology & Metabolism	2003	Not given	16	14–17	Israel	Crossover RCT	6	G, H, K, Q	DQOL-Y	Not given

*G: Hypoglycaemia; H: HbA1c; K: Ketoacidosis Q: Quality of life

**Randomised controlled trial

***Cross-sectional study

****Clinical trial

°Not applicable (cross-sectional study)

**Table 4:** Quality of life, HbA1c and adverse events data from all included studies.

					HbA1c (%)*		Quality of life**			Adverse events (per person year)***		
Lead author	Year	Follow-up period (months)	Treatment	Age group (years)	Baseline	Follow-up	QoL assessment tool	Baseline	Follow-up	Hypoglycaemia	Keto acidosis	Comments
**Mueller-Godeffroy [8]**	2018	6	CSII	6–7	7 (± 0.8)	7 (± 0.5)	KINDL-DM	Not given	Not given	Not given		-
				8–11	7.2 (± 0.8)	7.1 (± 1.0)		68.1 (± 14.9)	74.5 (± 12.0)			
				12–16	7.4 (± 1.1)	7.3 (± 1.0)		70.6 (± 11.9)	74.2 (± 13.0)			
			MDI	6–7	7.2 (± 0.8)	7.1 (± 0.7)		Not given	Not given			
				8–11	7.5 (± 1.1)	7.6 (± 1.1)		61.8 (± 15.2)	64.3 (± 14.9)			
				12–16	7.8 (± 1.5)	7.8 (± 1.3)		67.8 (± 16.9)	70.9 (± 16.0)			
**Blair [15]**	2018	12	CSII	0.6–4	11.7 (± 4.4)	8 (± 3.3)	PedsQL	Not given		0.0417	0.014	QoL only reported as medians and interquartile ranges (IQR)
				5–11		7.5 (± 3.2)						
				12–15		7.8 (± 3.4)						
			MDI	0.6–4	11.5 (± 4.6)	7.5 (± 3.1)				0.0134	0	
				5–11		7.6 (± 3.2)						
				12–15		7.2 (± 3.5)						
**Cherubini [38]**	2014	N/A****	CSII	10–17	N/A	Not given	IDSRQ	Not given		0.1332	0.0444	HbA1c reported as medians and IQRs only, no overall QoL scores given
			MDI							0.1176	0.0396	
**Birkebaek [36]**	2014	N/A	CSII	8–17	N/A	7.8 (not given)	PedsQL	N/A	81.2 (± 12.0)	Not given		Confidence intervals (CI) and standard errors not reported for HbA1c
			MDI			8.2 (not given)			79.9 (± 12.1)			
**Rendell [31]**	2013	N/A	CSII	9–17	N/A	8.1 (± 0.2)	Community Assessment Instrument Pre-Test 17, WHOQOL-BREF	N/A	Not given	Not given		No overall QoL scores given
			MDI			8.8 (± 0.5)						
**Lukacs [40]**	2013	N/A	CSII	8–18	N/A	8.6 (± 1.5)	PedsQL	N/A	82.1 (± 9.2)	Not given		-
			MDI			8.8 (± 1.6)			77.0 (± 10.0)			
**Wu [32]**	2010	N/A	CSII	12–17	N/A	8.2 (± 1.3)	DQOL-Y	N/A	77.3 (± 10.4)	Not given		-
			MDI			8.5 (± 2.0)			74.1 (± 11.5)			
**Mueller-Godeffroy [35]**	2009	6	CSII	4–7	Not given	7.3 (± 1.1) 7.4 (± 0.9) 7.6 (± 1.3)	KINDL-DM	Not given	79.9 (not given)	0.0172	Not given	CI and standard errors not reported for QoL in CSII group
				8–11					77.4 (not given)			
				12–16					76.3 (not given)			
			MDI	4–7		7.4 (± 1.4) 7.6 (± 0.8) 8.0 (± 1.6)			62.3 (± 11.7)	0.0172		
				8–11					64.2 (± 15.1)			
				12–16					69.6 (± 11.7)			
**Nuboer [39]**	2008	3.5	CSII	4–16	7.7 (± 0.6)	7.5 (± 0.5)	PedsQL	86.0 (± 9.5)	88.8 (± 9.0)	0.2900	0.2353	-
			MDI		8.0 (± 0.6)	8.0 (± 0.8)		81.9 (± 11.6)	82.3 (± 12.8)	1.1000	0.0714	
**Johannesen [37]**	2008	12	CSII	13–19	9.5 (± 1.6)	8.9 (not given)	Not specified (“validated QoL questionnaire”)	Not given		0.1333	0.4667	CI and standard errors not reported for HbA1c follow-up, no data on QoL reported
			MDI		9.7 (± 1.6)	9.5 (not given)				1.1000	0.0714	
**Wilson [33]**	2005	12	CSII	1–6	8.0 (± 0.8)	7.8 (not given)	DQOL for toddlers°	2.3 (± 0.3)	2.1 (not given)	0.1111	0	Confidence intervals and standard errors not reported for HbA1c and QoL follow-up
			MDI		8.0 (± 0.8)	8.0 (not given)		2.3 (± 0.6)	2.2 (not given)	0.1000	0	
**O'Neil [34]**	2005	N/A	CSII	9–17	N/A	7.5 (± 1.0)	DQOL-Y	N/A	Not given	Not given		No overall QoL scores given
			MDI			8.1 (± 1.5)						
**Shehadeh [41]**	2004	12	CSII	1–6	Not given	8.2 (± 0.9)	DQOL, modified°	Not given	33.7 (± 7.9)	0.2900	0	DQOL not comparable with DQOL-Y, QoL assessment after 4 months
			MDI			8.8 (± 1.0)			43.7 (± 8.0)	0.3600	0	
**Weintrob[42]**	2003	3.5	CSII	9–13	8.0 (± 1.1)	8.0 (± 0.7)	DQOL-Y	Not given		0.1300	0	No overall QoL scores given
			MDI		8.3 (± 0.7)	8.1 (± 0.8)				0.3900	0	
**Cohen[43]**	2003	6	CSII	14–17	8.6 (± 0.8)	8.2 (± 1.3)	DQOL-Y	Not given		0.0830	0.0833	No overall QoL scores given
			MDI		8.5 (± 1.4)	8.6 (± 0.4)				0.3330	0	

* Mean HbA1c and standard deviation (when given)

** Mean values per person year. If incidence was reported, sample sizes and follow-up time were used to calculate rates per person years.

*** Mean overall scores and standard deviation (when given). Higher scores indicate higher QoL except for tools marked with° where lower scores indicate a higher QoL

**** No reporting of follow-up time and baseline data due to cross-sectional study design

**KINDL-DM**: specific diabetes module of KINDL-R (not used as acronym)

**PedsQL**: Pediatric Quality of Life Inventory

**IDSRQ**: Insulin Delivery System Rating Questionnaire

**WHOQOL-BREF**: World Health Organization Quality of Life Questionnaire (short version)

**DQOL-Y**: Diabetes Quality of Life for Youth Questionnaire

**DQOL**: Diabetes Quality of Life Questionnaire

### Quality of life

**Fig 2:**
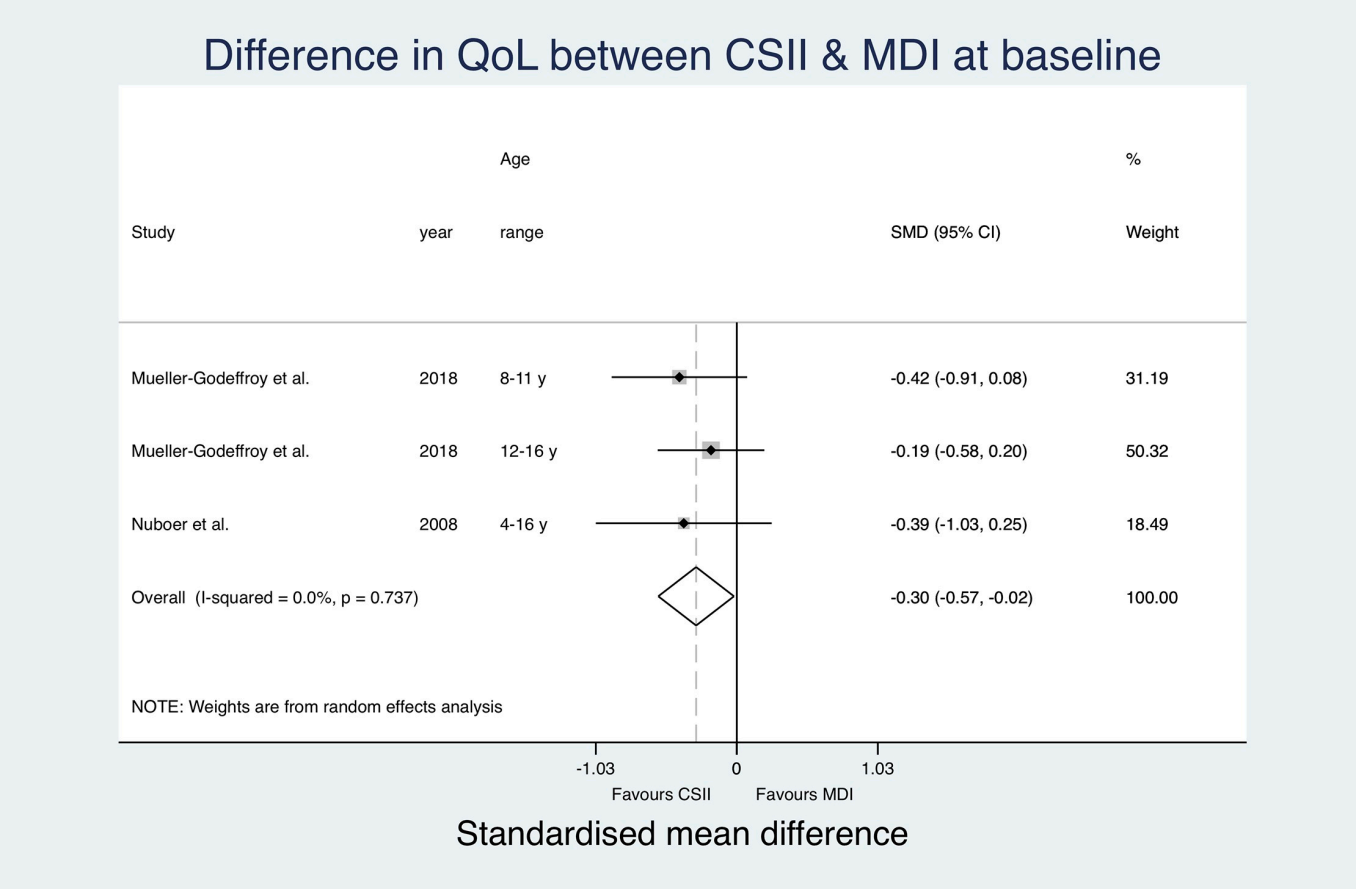
Pooled quality of life results (SMD) at baseline.

**Fig 3:**
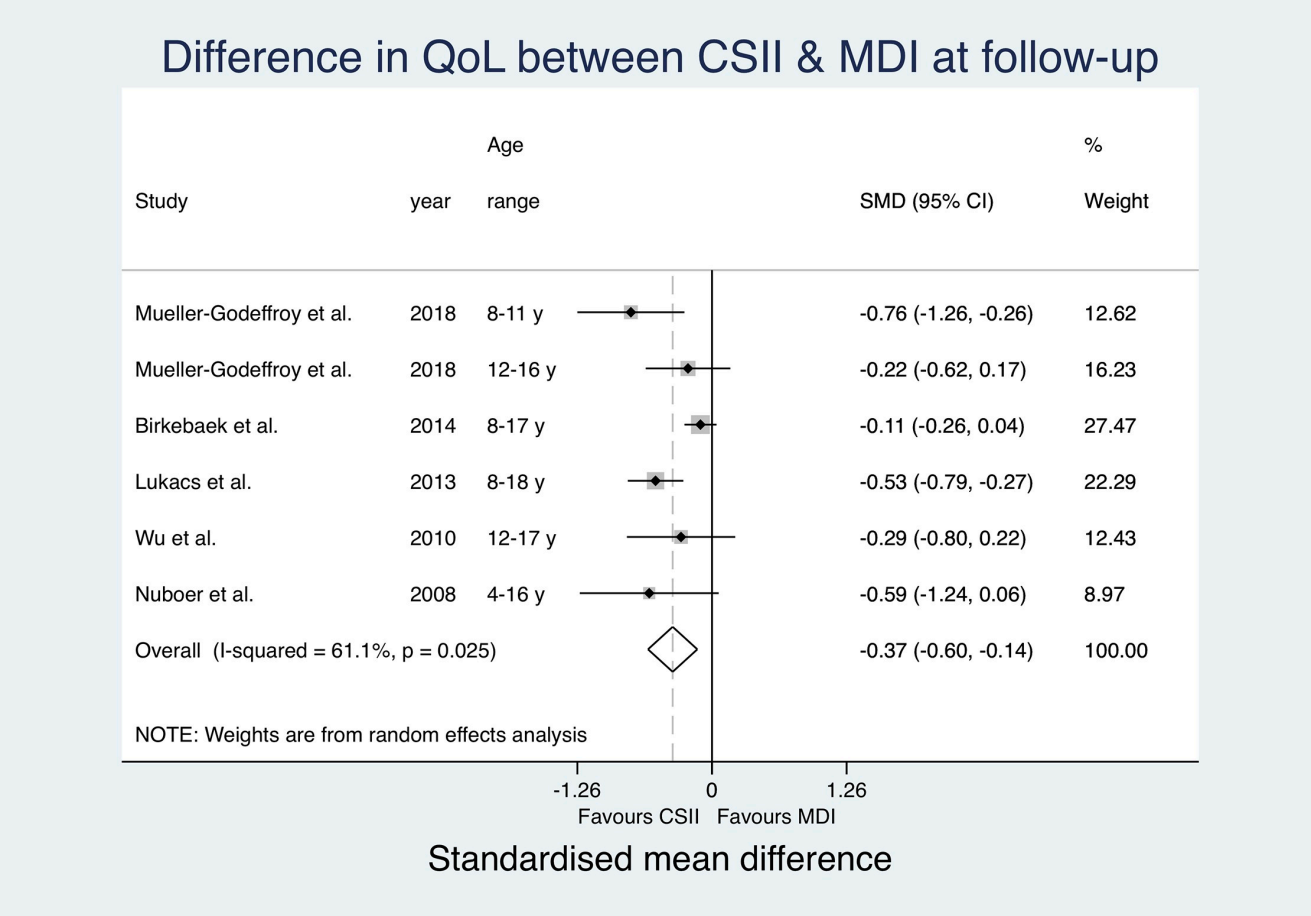
Pooled quality of life results (SMD) at follow-up.

All included studies reported QoL measures using eight different assessment tools (Table 4). Both studies by Mueller-Godeffroy et al. used the KINDL-DM[8, 35], the Pediatric Quality of Life Inventory (PedsQL)[15, 36, 39, 40] and the Diabetes Quality of Life for Youth Questionnaire (DQOL-Y)[32, 34, 42, 43] were used in four papers respectively. Cherubini et al. assessed QoL using the Insulin Delivery System Rating Questionnaire (IDSRQ)[38], Rendell et al. used the World Health Organization Quality of Life Questionnaire (WHOQOL-BREF)[31] and Johannesen et al. did not state the name of the applied questionnaire.[37] Two studies used modified versions of the Diabetes Quality of Life Questionnaire (DQOL).[33, 41] Three studies reported both QoL at baseline and at follow-up.[8, 33, 39] Six studies reported significantly higher QoL in the CSII group compared to MDI subjects,[8, 35, 38, 40, 41, 43] three studies reported that QoL in the CSII cohort was higher but the difference did not reach significance level.[15, 31, 36] Six papers could not find a difference between QoL in MDI and CSII users.[32–34, 37, 39, 42] Due to large heterogeneity in QoL assessment tools and differences in reporting of QoL (e.g. missing CIs, reporting of medians instead of means), only SMD for baseline data from two studies[8, 39] (one study reporting separately for two age groups)[8] and SMD for follow-up data from five studies[8, 32, 36, 39, 40] (one study reporting separately for two age groups again)[8] could be pooled for an overall effect estimate using a random effects meta-analysis (Fig 2 and Fig 3).

The effect estimates at baseline do not suggest significantly relevant heterogeneity (I^2^ = 0.0%). The pooled estimates show a significant overall SMD in QoL favouring the CSII group (Overall SMD -0.3 (-0.02, -0.57); p = 0.035).

At follow-up, the entered effect estimates suggest substantial heterogeneity (I^2^ = 61.1%) between studies. The pooled estimates suggest significantly better QoL in the CSII group (Overall SMD -0.37 (-0.14, -0.60); p = 0.002). The pooled estimated SMD in QoL between study groups was more prominent at follow-up, but still only showing a small difference. Changes between baseline and follow-up over time did not reach significance level. CSII groups reported significantly better QoL at both measurements.

### Adverse events

Nine studies included adverse events in their results (Table 4).[15, 33, 35, 37–39, 41–43] Eight studies reported numbers for both severe hypoglycaemia and ketoacidosis with solely Mueller-Godeffroy et al.[35] reporting numbers for hypoglycaemia only. Eight studies did not suggest any significant differences in adverse events between treatment groups.[15, 33, 35, 37, 38, 41–43] Nuboer et al. did show a threefold decrease of hypoglycaemia incidence in CSII subjects but did not state significance of the result. Also, no differences in ketoacidosis incidence were found. The performed Mann-Whitney U test did neither show any significant difference in severe hypoglycaemia rates (p = 0.2888) nor in ketoacidosis rates (p = 0.1052) between treatment groups (Table 5 & Table 6). Results suggest that incidence rates per patient year for severe hypoglycaemia were slightly higher with MDI. However, incidence rates for ketoacidosis proved to be higher with CSII treatment.

**Table 5:** Mann-Whitney U test for comparison of hypoglycaemia rates between treatment groups.

Treatment group	Observations	Rank sum	Expected
**CSII**	9	73.5	85.5
**MDI**	9	97.5	85.5
**Combined**	18	171	171
**H0: Hypoglycaemia CSII = Hypoglycaemia MDI** **z = -1.061** **Prob > |z| = 0.2888**			

**Table 6:** Mann-Whitney U test for comparison of ketoacidosis rates between treatment groups.

Treatment group	Observations	Rank sum	Expected
**CSII**	8	82	68
**MDI**	8	54	68
**Combined**	16	136	136
**H0: Ketoacidosis CSII = Ketoacidosis MDI** **z = 1.620** **Prob > |z| = 0.1052**			

### HbA1c

**Fig 4:**
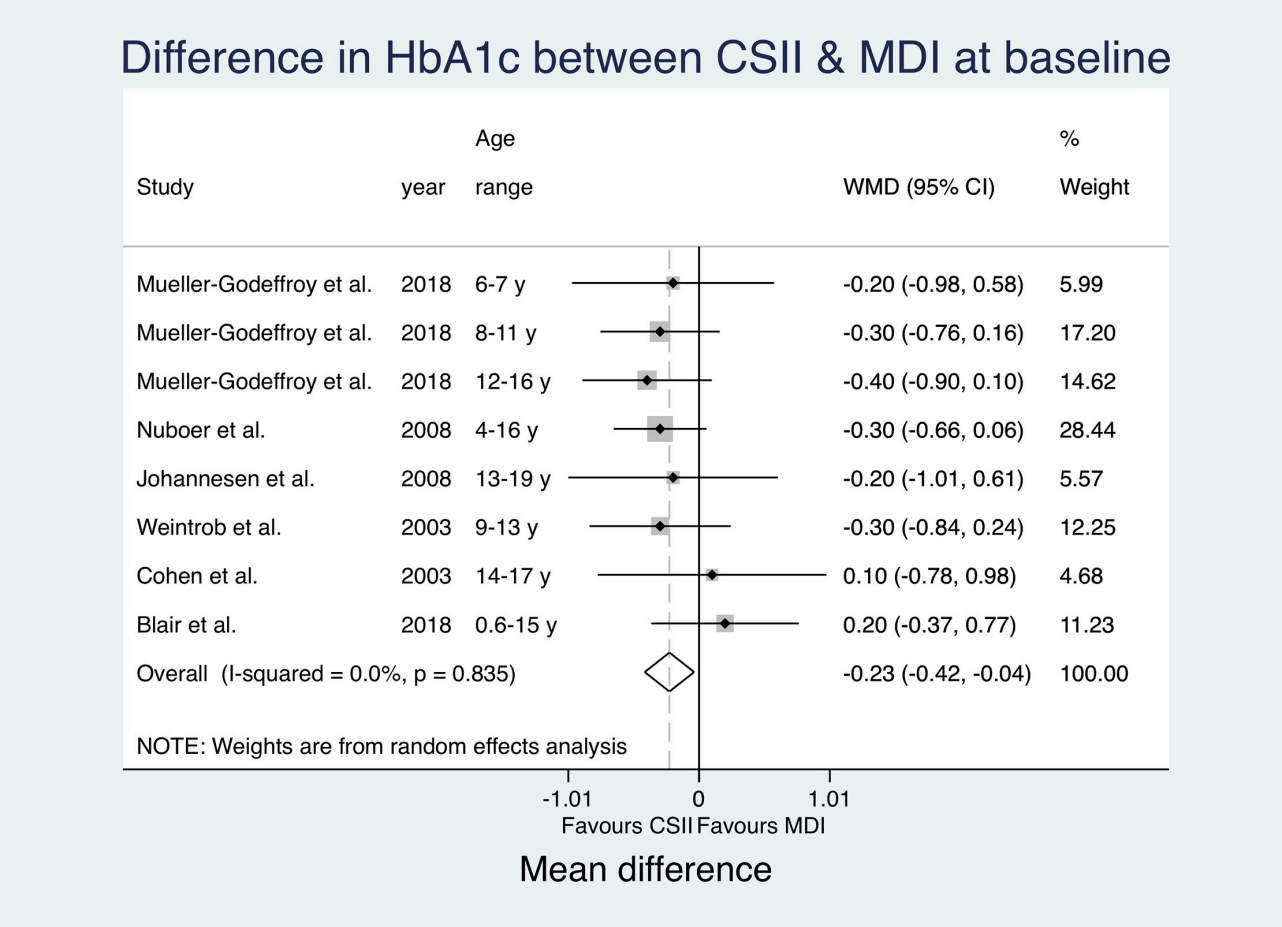
Pooled HbA1c results (MD) at baseline.

**Fig 5:**
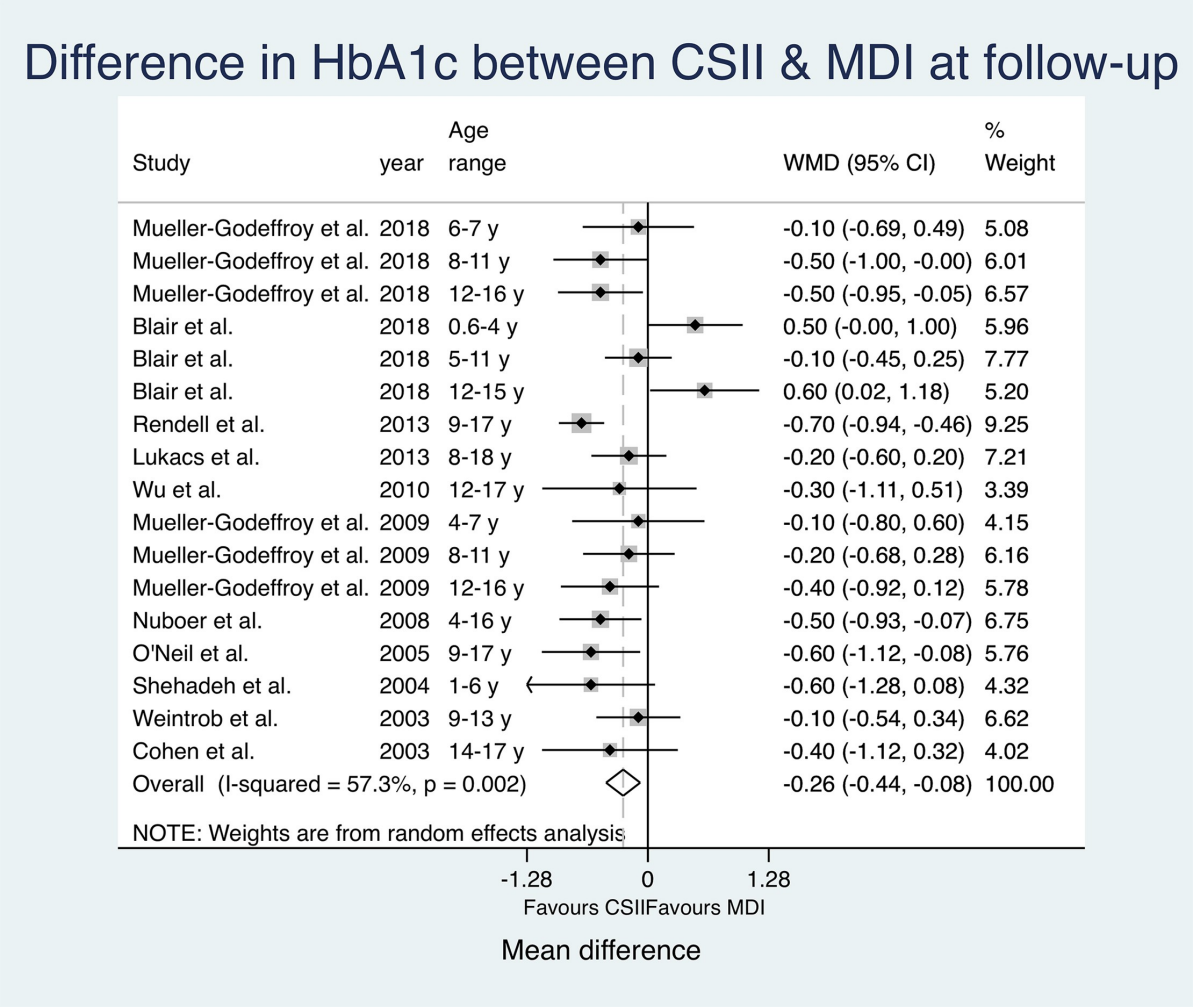
Pooled HbA1c results (MD) at follow-up.

HbA1c measures were reported by all studies (Table 4). Seven studies included baseline data[8, 15, 33, 37, 39, 42, 43] and one study did not report numbers for HbA1c at follow-up.[38] Five papers found significantly lower HbA1c levels in CSII subjects.[34–36, 39, 41] However, one study only reported significant results for one age group.[35] Ten studies did not find significant differences between treatment groups.[8, 15, 31–33, 37, 38, 40, 42, 43] All but two studies[15, 38] reported better HbA1c measures for CSII. Cherubini et al. did not state any numbers and Blair et al. did report better HbA1c outcomes for one of the three examined age groups only. Due to differences in reporting of HbA1c (e.g. missing CIs, reporting of medians instead of means, etc.), only MD for baseline data from six studies[8, 15, 37, 39, 42, 43] (one study reporting separately for three age groups)[8] and MD for follow-up data from eleven studies[8, 15, 31, 32, 34, 35, 39–43] (three studies reporting separately for three age groups again)[8, 15, 35] could be pooled for an overall effect estimate using a random effects meta-analysis (Fig 4 and Fig 5).

The effect estimates at baseline do not suggest significantly relevant heterogeneity (I^2^ = 0.0%). The pooled estimates show a significant overall mean difference in HbA1c favouring CSII (Overall MD -0.23 (-0.42, -0.04); p = 0.019). The calculated effect estimates at follow-up suggest substantial heterogeneity (I^2^ = 57.3%) between studies. Again, pooled estimates show a significant mean difference between groups favouring CSII (Overall MD -0.26 (-0.44, -0.08); p = 0.005).

The estimated mean difference in HbA1c between study groups was bigger at follow-up. CSII groups reported lower HbA1c at both measurements with an even more significant difference between study groups at follow-up. However, changes over time did not reach significance level.

The results of the Funnel Plots and Egger test to evaluate publication bias in HbA1C at baseline and follow and QoL, showed only an asymmetrical appearance in the HbA1C at baseline (please see supplementary material for all analyses). Assuming that studies with high precision should be plotted near the average, and those with low precision should be spread evenly on both sides of the average, one can clearly see that in the case of HbA1C at baseline the distribution of studies tends to be concentrated towards a value of 0 which is suggestive of certain publication bias.

## Discussion

The current meta-analysis could show significant differences in QoL between paediatric CSII and MDI users at follow-up. However, QoL was better in CSII subjects at baseline, too, which is a bias potentially mitigating the validity of the performed analysis. No significant change in QoL over time could be observed. The evidence suggested no significant differences in adverse event incidence between treatment groups. Severe hypoglycaemia incidence was higher in MDI subjects whereas ketoacidosis incidence was shown to be higher in CSII subjects. A significant difference in glycaemic control could be shown between treatment groups both, at baseline and follow-up, with CSII yielding lower HbA1c values at both points in time respectively. Despite the difference being more prominent at follow-up, no significant change could be shown over time.

Several limitations of the included literature have to be considered when interpreting the results of this work. Specific methodological issues are described in Table 7. The described heterogeneity is in part reflected in statistical results of the meta-analysis with both the pooled follow-up estimates of QoL and HbA1c analyses showing substantial and considerable heterogeneity respectively. In addition to limitations of the included literature there are also relevant limitations to this review itself (Table 8). Due to the small number of studies included and the heterogeneity in study design and methodology, the appropriateness of a meta-analysis has to be critically questioned and the results of this review should be seen in the context of the stated limitations.

**Table 7:** Limitations of the included literature.

Limitation	Potential issue
**Differences in study design**	- No exclusion criteria were defined regarding study design - → Inclusion of six RCT[8, 15, 33, 39, 42, 43] (strongest design when assessing primary data[44]) as well as several other study types (CSS, CT, prospective observational) - The latter do not include randomisation and thus, cannot rule out whether differences in outcome are caused by the exposure or influenced by differences in other observed and unobserved characteristics between groups - → Possible introduction of systematic error
**Differences in sample size**	- Eight of the studies[31–33, 37, 39, 41–43] included less than 100 patients with two studies reporting for only fifteen[41] and sixteen[43] patients respectively - → With small sample sizes, observed results are more likely to be caused by chance and might not be representative of the population
**Differences in study location**	- Only studies from the USA, Europe and Israel were included, recruiting patients from one to eighteen different study sites within the respective countries - → Potential source of selection bias
**Differences in primary data source**	- One study used a national registry as data source[36] - Three studies[31, 34, 40] recruited their participants from diabetes summer camps - → Potential source of selection bias
**Differences in age**	- Age groups differed between studies (Table 3) - → Parents answered questionnaires as proxies for younger children (Table 9) which might mitigate the comparability between studies
**Differences in adjustment for confounders**	- No consistent reporting (Table 3) - → Six studies did not state any adjustment[31–33, 35, 41, 43] and thus, existence of residual, unaddressed and unidentified confounding throughout studies cannot be ruled out

**Table 8:** Limitations of the review.

Limitation of review	Potential issue
**Number of databases**	- Only three databases were used for literature search - → Potential selection bias which could mitigate representativeness of the results
**Accessibility of studies**	- Full-texts could not be accessed for twelve possibly relevant studies - Search was limited to titles and abstracts in English only - → Potential selection bias
**Differences in reporting**	- Two studies reported medians and IQR for HbA1c only,[15, 38] five studies did not report overall QoL scores[31, 34, 38, 42, 43] and four studies did neither report CI nor standard error[33, 35–37] - → Only a very small number of studies was included in the analyses which mitigates the generalisability of the results and increases the probability of them occurring by chance alone
**Publication bias**	- Studies reporting significant or interesting results are more likely to be published - → The findings of this review are likely to be affected by publication bias.
**Registration**	- This paper has not been registered through PROSPERO prior to publication - → The risk of other reviews addressing the same question being published simultaneously cannot be ruled out

Huge heterogeneity could be found regarding case ascertainment (Table 9). The quality of studies was assessed using the EPHPP quality assessment tool for quantitative studies (S1 Table). The tool generates a total quality score between one (strong) and three (weak) based on six sub-scores, assessing components of internal and external validity.[22] All but two of the studies were awarded three points.[8, 35] Most papers were rated weak because of a lack in blinding (due to the nature of the exposure) and not mentioning validity and reliability of the applied assessment tools for QoL. In addition, all of the included CSS were awarded weak component ratings for study design. Overall, the evidence base is rated as weak. However, the applied tool might not be ideal for rating the quality of studies on insulin treatment regimens since some of the assessed components seem to be inappropriate (e.g. blinding). Also, the tool does not address and rate patient reported outcome measures like QoL or treatment satisfaction specifically.

**Table 9:** Exposure and outcome ascertainment for included studies.

	Differences in case ascertainment	
**Exposure**	Diabetes duration prior to study	- Minimum duration of diabetes before study entry ranged between six months and two years for ten studies, was not defined for four studies[31, 33, 34, 36] and Blair et al. only included newly diagnosed children
	Follow-up time	- Follow-up times ranged from 3.5 to 12 months in length with six studies reporting no times due to their cross-sectional design[31, 32, 34, 36, 38, 40]
	Insulin delivery	- The same well-established MDI and CSII definitions were applied throughout studies - Insulin and delivery system types varied greatly between studies
**Outcome**	QoL	- The fifteen studies used eight different QoL assessment tools (Table 4) with one study not specifying the used tool[37] - Parents answered questionnaires for children in younger age groups[8, 15, 33, 35, 39, 41] - Four studies did not report overall QoL scores[31, 34, 42, 43] - Baseline data on QoL could only be included for three studies[8, 33, 39]
	Adverse events	- Only nine out of fifteen studies reported results on adverse events - Results were presented in events per person time[38, 39, 41–43] as well as crude number of events per treatment group[15, 33, 35, 37]
	HbA1c	- Fourteen studies reported HbA1c in % whereas Blair used mmol/mol - Different HbA1c analysers were used throughout studies with some CSS using the latest HbA1c values reported by caregivers or retrieved from patient records

Nowadays, the biggest challenge in paediatric diabetes therapy is reaching optimal glycaemic control while maintaining the highest possible QoL for children and their parents or caregivers.[8] Different aspects seem to be of importance for reaching this goal. Positive relationships as well as good glycaemic control and sufficient self-management have been shown to substantially decrease the burden DM inflicts on the affected individuals.[18] In this respect, recent technological developments in insulin application methods like CSII systems have improved insulin therapy through providing more flexibility.[45] Through CSII paediatric patients can carry out precise insulin dose adjustments and have greater independence and more responsibility.[46] These attributes are considered main benefits of CSII.[47] However, recent reviews solely showed that QoL in T1DM children and adolescents using CSII is slightly higher or similar to QoL in MDI patients.[18, 19] In contrast, Blair et al. stated in 2018 that CSII is neither cost-effective nor clinically superior to MDI.[15] Partly aligned with this, this analysis could in fact show significant differences in QoL at both, baseline and follow-up but no significant change over time could be observed. Having said that, glycaemic control proved to be considerably better in CSII patients but no change over time could be seen. In addition to QoL and glycaemic control, acute complications associated with CSII need to be addressed since these are major concerns for patients when transitioning to CSII.[18] Most studies with short follow-up periods did not show any difference in adverse events between treatment groups whereas studies with follow-up times of more than one year showed decreased adverse event rates for CSII patients.[18] This review concurs previous findings. However, due to the follow-up times of the included evidence, no long-term effects of CSII on morbidity and mortality in T1DM patients could be assessed. This proves an important limitation since chronic complications of T1DM can impose an enormous additional burden on patients’ lifestyle with detrimental effects to their QoL. Due to several methodological limitations and vast heterogeneity in the included evidence, recommendations based on this review should be considered with due care. We could not show considerable superiority of CSII over MDI regarding QoL in paediatric patients. Thus, it cannot be recommended to replace MDI as first-choice treatment in T1DM youth. However, since outcomes regarding QoL and glycaemic control could be shown to be similar to MDI and decreased mortality because of long-term complications in CSII patients has been described in previous studies[48], public health professionals should reconsider including CSII as an additional first-line treatment in T1DM equal to MDI.

## Conclusion

This paper set out to analyse whether CSII regimens are superior to MDI therapy in T1DM youth regarding HRQOL. Additionally, it assessed glycaemic control and adverse events as secondary outcomes and discussed potential future public health implications and justifications for using CSII as a first-line therapy in children and adolescents. Despite the stated limitations and the fact that no considerable difference in QoL between treatment groups could be shown over time, CSII proved to provide similar or slightly better outcomes in all analysed fields. This is consistent with previous research. However, to make credible and reliable recommendations, bigger, better-powered and better-designed studies are needed to investigate the impact of CSII in children. Poor methodology, small samples and short follow-up times constrain the ability to assess the association between QoL and CSII to the full extent.[17]

## Supporting information

S1 Table Quality assessment of included literature using the EPHPP.[23]. (DOCX)

S1 File PRISMA checklist. (DOCX)

S2 File Protocol for systematic review and meta-analysis. (DOCX)

S1 Fig Pooled quality of life results (SMD) at baseline by study type. Sensitivity analysis.
(TIFF)

S2 Fig Pooled quality of life results (SMD) at follow-up by study type. Sensitivity analysis.
(TIFF)

S3 Fig Pooled HbA1c results (MD) at baseline by study type. Sensitivity analysis.
(TIFF)

S4 Fig Pooled HbA1c results (MD) at follow-up by study type. Sensitivity analysis.
(TIFF)

S5 Fig Funnel plot for pooled quality of life results at baseline. (TIFF)

S6 Fig Egger’s regression test for pooled quality of life results at baseline. (TIFF)

S7 Fig Funnel plot for pooled quality of life results at follow-up. (TIFF)

S8 Fig Egger’s regression test for pooled quality of life results at follow-up. (TIFF)

S9 Fig Funnel plot for pooled HbA1c results at baseline. (TIFF)

S10 Fig Egger’s regression test for pooled HbA1c results at baseline. (TIFF)

S11 Fig Funnel plot for pooled HbA1c results at follow-up. (TIFF)

S12 Fig Egger’s regression test for pooled HbA1c results at follow-up. (TIFF)

## Abbreviations

ADA: American Diabetes Association
AFR: Africa
BMI: Body mass index
CI: Confidence interva
CSII: Continuous subcutaneous insulin infusion
CSS: Cross-sectional study
CT: Controlled trial
DM: Diabetes mellitus
DQOL: Diabetes Quality of Life Questionnaire
DQOL-Y: Diabetes Quality of Life for Youth Questionnaire
EPHPP: Effective Public Health Practice Project Tool
EUR: Europe
HbA1c: Glycated haemoglobin
HRQOL: Health-related quality of life
IDF: International Diabetes Federation
IDSRQ: Insulin Delivery System Rating Questionnaire
IQR: Interquartile range
MDI: Multiple dose injection therapy
MENA: Middle East and North Africa
N/A: Not applicable
NAC: North America and Caribbean
NICE: National Institute for Health and Care Excellence
PedsQL: Pediatric Quality of Life Inventory
RCT: Randomised controlled trial
SACA: South and Central America
SEA: South-East Asia
SMD: Standardised mean difference
T1DM: Type 1 Diabetes mellitus
T2DM: Type 2 Diabetes mellitus
UK: United Kingdom
US: United States (of America)
USD: United States-Dollar
QoL: Quality of life
VO_2_max: Maximum rate of oxygen consumption during exercise
WHO: World Health Organization
WHOQOL-BREF: World Health Organization Quality of Life Questionnaire (short version)
WP: Western Pacific
