# Supplementary material for: Health-related quality of life in paediatric patients with Type 1 diabetes mellitus using insulin infusion systems. A systematic review and meta-analysis
Source: PLoS One. 2019 Jun 25;14(6):e0217655. doi: 10.1371/journal.pone.0217655 (PMC6592525; doi:10.1371/journal.pone.0217655)
Supplement: S1 Table — (DOCX) [file pone.0217655.s001.docx]

**Table 10:** Quality assessment of included literature using the EPHPP^23^

| Citation | Mueller-G.^8^ (2018) | Blair^15^ | Cherubini^38^ | Birkebaek^36^ | Rendell^31^ | Lukacs^40^ | Wu^32^ | Muller-G.^35^ (2009) | Nuboer^39^ | Johannesen^37^ | Wilson^33^ | O'Neil^34^ | Shehadeh^41^ | Weintrob^42^ | Cohen^43^ |
| --- | --- | --- | --- | --- | --- | --- | --- | --- | --- | --- | --- | --- | --- | --- | --- |
| Study design | RCT* | RCT | CSS** | CSS | CSS | CSS | CSS | Prospective observational  study | Parallel RCT | CT*** | RCT | CSS | Crossover CT | Crossover RCT | Crossover RCT |
| Sample source | 18 diabetic centres in Germany | 15 diabetic clinics in England & Wales | 14 Italian paediatric diabetic centres | Families registered in 2009 in the Danish Registry for Childhood Diabetes | one diabetes summer camp, Eastern Nebraska, US | diabetes summer camps in Hungary | diabetes clinics, US; not specified | 18 diabetic centres in Germany | Different sites in the Netherlands, not specified | one hospital and one diabetes clinic, Denmark | two diabetes centres, US | one diabetes camp, US | two paediatric diabetes clinics in Israel and one in Slovenia | one paediatric diabetes clinic, Israel | one paediatric diabetes clinic, Israel |
| Number of subjects | 211 | 293 | 577 | 700 | 53 | 239 | 62 | 117 | 38 | 56 | 19 | 103 | 15 | 23 | 16 |
| A. Selection Bias | 1 | 3 | 2 | 2 | 2 | 2 | 3 | 1 | 2 | 2 | 2 | 3 | 3 | 2 | 3 |
| B. Design | 1 | 1 | 3 | 3 | 3 | 3 | 3 | 2 | 1 | 1 | 1 | 3 | 1 | 1 | 1 |
| C. Confounders | 1 | 1 | 1 | 2 | 1 | 1 | 1 | 1 | 1 | 1 | 3 | 1 | 1 | 1 | 1 |
| D. Blinding | 3 | 3 | 3 | 3 | 3 | 3 | 3 | 3 | 3 | 3 | 3 | 3 | 3 | 3 | 3 |
| E. Data collection methods | 1 | 3 | 1 | 3 | 3 | 3 | 1 | 1 | 3 | 2 | 3 | 1 | 3 | 3 | 3 |
| F. Withdrawals & Dropouts | 1 | 1 | N/A**** | N/A | N/A | N/A | N/A | 2 | 1 | 3 | 2 | N/A | 1 | 1 | 2 |
| Overall score | **1** | **3** | **3** | **3** | **3** | **3** | **3** | **2** | **3** | **3** | **3** | **3** | **3** | **3** | **3** |

**Quality indicators based on the EPHPP^48^** * Randomised controlled trial

1 = “strong” rating of component; 2 = “moderate” rating of component; 3 = “weak” rating of component; N/A = Only one time point analysed ** Cross-sectional study
**Overall score based on** *** Clinical trial

1 = Strong (no “weak” ratings); 2 = Moderate (one “weak” rating); 3 = Weak (two or more “weak” ratings) **** Not applicable
