# Supplementary material for: Health-related quality of life in paediatric patients with Type 1 diabetes mellitus using insulin infusion systems. A systematic review and meta-analysis
Source: PLoS One. 2019 Jun 25;14(6):e0217655. doi: 10.1371/journal.pone.0217655 (PMC6592525; doi:10.1371/journal.pone.0217655)
Supplement: S2 File — (DOCX) [file pone.0217655.s003.docx]

Version 3, March 2011

Systematic Review Protocol & Support Template*

* Procotol template retrieved from Prospero (International prospective register of systematic reviews; www.crd.york.ac.uk)

| **Title of the review** | Health-related quality of life in paediatric patients with Type 1 Diabetes Mellitus using insulin infusion systems.  A systematic review and meta-analysis. |
| --- | --- |
| **First reviewer** | Bastian Rosner |
| **Team of reviewers** | Andres Roman-Urrestarazu MD PhD |
| **Supervisor/Project PI** | Andres Roman-Urrestarazu MD PhD |
| **Clinical Portfolio Group** | - |
| **Project title (if different from review title)** | - |

| **Support** – please state if advice/training or personnel required at each stage | |
| --- | --- |
| **SR overview** | Bastian Rosner & Andres Roman-Urrestarazu |
| **Protocol development** | Bastian Rosner & Andres Roman-Urrestarazu |
| **Literature searching** | Already had training from library on literature searching and reference management as part of postgraduate studies |
| **Quality appraisal** | Bastian Rosner |
| **Data Extraction** | Bastian Rosner & Andres Roman-Urrestarazu |
| **Synthesis** | Bastian Rosner |

| **1. Background to review**  Brief introduction to the subject of the review, including rationale for undertaking the review and overall aim |
| --- |
| Diabetes mellitus (DM) is one of the top ten causes of global mortality, having killed 1.6 million people in 2016 alone (WHO, 2016 & 2018). DM describes a cluster of metabolic diseases, rather than a single illness, that are characterised by chronic hyperglycaemia (Amboss, 2019). The American Diabetes Association (ADA) classifies DM into four general categories with the most common ones being type 2 diabetes mellitus (T2DM) and type 1 diabetes mellitus (T1DM) following in second place (Amboss, 2019; ADA, 2015). It is estimated that more than 96,000 children under the age of fifteen are diagnosed with T1DM annually, whilst there are 1.1 million children and adolescents below 20 years living with T1DM globally (IDF, 2017). There are considerable regional differences in the prevalence of T1DM with more than one quarter (28.4%) of paediatric patients living in Europe and more than one fifth (21.5%) living in North America and the Caribbean. The highest incidence of T1DM can be seen in the United States (US), India and Brazil (IDF, 2017). Complications in T1DM are relatively frequent and can be divided into acute (e.g. diabetic ketoacidosis, infection, polydipsia, fatigue) and chronic (macro- and microangiopathy). In addition to being a global health problem due to its multiple short and long-term complications, diabetes and related conditions account for an enormous economic burden throughout the world (Bommer et al, 2017). This burden is expected to continue growing with a projected expenditure of 776 billion US-Dollar by 2045 for adult patients only (IDF, 2017). T1DM’s physiopathology is primarily due to β-cell destruction and absolute insulin deficiency (Amboss, 2019). Thus, the therapeutic goal for T1DM patients is defined as reaching optimal glycaemic control as early as possible to avoid acute and chronic complications without compromising the quality of life (QoL) and wellbeing of children, their parents or caregivers (Mueller-G. et al, 2018). The only way to reach this goal for patients with T1DM is - additionally to behavioural interventions - the uninterrupted supply of insulin (IDF, 2017). Insulin regimens available for T1DM patients can be divided into three groups: multiple daily injection basal-bolus insulin regimens (MDI), mixed (biphasic) regimens and continuous subcutaneous insulin infusion regimens (CSII, insulin pump). Despite MDI still being the first-line therapy in many regions around the world (Silver et al, 2018; NICE, 2015; ADA, 2018), CSII is gaining popularity among paediatric patients (Sherr et al, 2016). This can be explained to some extend by slightly better metabolic control and less acute complications through CSII (Sherr et al, 2016; Misso et al, 2010; Karges et al, 2017) but might also be influenced by other factors not yet fully understood (Mueller-G. et al, 2018). With CSII being much more expensive than MDI – treatment cost would increase by 50% if all T1DM patients used CSII15 – methodologically well-conducted studies are needed to prove its superiority over MDI and to justify it as a first-line choice.  So far, reviews investigating insulin pump therapy showed mixed results regarding the health-related quality of life (HRQOL) in paediatric diabetes patients (Misso et al, 2010; Yeh et al, 2012; Barnard et al, 2007). According to recent publications (Yeh et al, 2012; Hirose et al 2012; Phillip et al, 2007; Golden et al, 2012) there is still a lack of adequately powered studies to underpin the advantages of CSII regarding QoL improvement for children diagnosed with DM and to potentially balance the higher treatment cost attached to it (Mueller-G. et al, 2018). Thus, insulin pumps are – forty years after they were first introduced to the market – still not part of first-line recommendations in most countries around the world.  Aim  The aim of this systematic review and meta-analysis is to analyse available evidence on whether CSII is superior to MDI therapy in T1DM youth regarding HRQOL. In addition, this work will assess glycaemic control and adverse events as secondary outcomes, since a close relationship between HRQOL and glycaemic control has been previously described. A thorough understanding of the links between both outcomes could have important implications for the adoption of CSII in paediatric diabetes care (Hirose et al, 2012). Finally, the paper will discuss the potential future public health significance and whether there is justification for using CSII as a first-line therapy in children and adolescents. |

| **2. Specific objectives** |
| --- |
| 1. To clarify and evaluate the evidence base regarding QoL in T1DM youth using CSII regimens. This will be done by conducting a systematic review and meta-analysis of the evidence base of journals and abstracts on this topic. T1DM youth on MDI regimens will serve as a reference group 2. To assess glycaemic control in T1DM children and adolescents on MDI and CSII regimens by comparing HbA1c measurements as proxy for glycaemic control 3. To compare incidence of adverse events between MDI and CSII treatment groups 4. To discuss potential public health significance of the findings and whether there is justification for using CSII as a first-line therapy in T1DM youth |

| **3. a) Criteria for including studies in the review**  If the PICOS format does not fit the research question of interest, please split up the question into separate concepts and put one under each heading | |
| --- | --- |
| 1. **Population, or participants and conditions of interest** | T1DM children in adolescents (19 years or younger) using insulin pumps (CSII), any gender, any country |
| 1. **Interventions or exposures** | T1DM youth using CSII regimens |
| 1. **Comparisons or control groups** | T1DM youth using MDI regimens |
| 1. **Outcomes of interest** | Quality of life (primary outcome); glycaemic control (HbA1c) and adverse event incidence as secondary outcomes) |
| 1. **Setting** | Outpatient setting |
| 1. **Study designs** | Any study design for primary data analysis: would expect to be observational/cohort studies as well as RCT’s |

| **3. b) Criteria for excluding studies not covered in inclusion criteria** Any specific populations excluded, date range, language, whether abstracts or full text available, etc. |
| --- |
| - Studies comparing quality of life between CSII regimens and control groups other than MDI (e.g. healthy controls, other pump regimens) - Studies solely referring to closed-loop systems or sensor-augmented pump therapy - Studies focused on type 2 diabetes mellitus (T2DM) specifically - Studies not primarily assessing quality of life - No restriction to children and/or adolescents (WHO definition) - Full-text not accessible at University of Cambridge or University of Groningen - Languages other than English - Review, meta-analysis |

| **4. Search methods** | |
| --- | --- |
| **Electronic databases**  Please list all databases that are to be searched and include the interface (eg NHS, EBSCO, etc) and date ranges searched for each | - PUBMED/MEDLINE - University of Cambridge Web of Science - Cochrane Library |
| **Other methods used for identifying relevant research**  ie contacting experts and reference checking | Reference checking and hand searching of these. Manually searching google.com for grey literature |
| **Journals hand searched**  If any are to be hand searched, please list which journals and date searched from, including a rationale. | - |

| **5. Methods of review** | |
| --- | --- |
| **Details of methods**  Number of reviewers, how agreements to be reached and disagreements dealt with, etc. | Two levels of screening by three independent researchers are used on all citations. Bastian Rosner as first reviewer, Andres Roman-Urrestarazu as second reviewer. If there will be any doubt, the article will be retained for the next level of scrutiny. Disagreements are resolved by face-to-face discussion, leading to a consensus judgement. |
| **Quality assessment**  Tools or checklists used with references or URLs | This protocol will define the method of literature appraisal used. The Effective Public Health Practice Project Tool (EPHPP) will be used to assess and compare the quality of included studies. |
| **Data extraction**  What information is to be collected on each included study. If databases or forms on Word or Excel are used and how this is recorded and by how many reviewers | The data extracted from each study will comprise lead author, sample characteristics, study setting, study design, follow-up details, information on exposure and outcome measurements as well as on confounders. Means will be used as the main measures of association across studies Data extraction form in Word document; Endnote to be used to keep track of references; Reviewer number 1 (BR) will review first, followed by reviewer number 2 (ARU), which will be done independently. Disagreements will be resolved by face-to-face discussion. |
| **Narrative synthesis**  Details of what and how synthesis will be done | Narrative synthesis of the data will be done and will be carried out addressing the following elements:   1. Results on case ascertainment and general reporting of each study 2. Results on QoL, analysing and comparing baseline as well as follow-up data 3. Results on adverse events, analysing and comparing incidence rates between groups 4. Results on HbA1c (glycaemic control), analysing and comparing baseline as well as follow-up data 5. Summary of results 6. Assessing the robustness of the synthesis and the evidence base |
| **Meta-analysis**  Details of what and how analysis and testing will be done. If no meta-analysis is to be conducted, please give reason. | To analyse the association between QoL and insulin treatment regimen, standardised mean differences (SMD) will be calculated for each study at baseline & at follow-up and will be entered into the random effects meta-analysis as primary effect measures. A Mann-Whitney U test will be carried out to look for potential associations between adverse event rates per patient year and the assigned treatment groups. For HbA1c mean differences (MD) between treatment groups at baseline and follow-up will be retrieved from each paper and entered into a random effects meta-analysis to approximate an overall pooled effect size for each point in time. Random effects models will be chosen for all meta-analyses because of anticipated between-study variance. Heterogeneity will be judged by using the Cochran Q test and the I2 statistic. |
| **Grading evidence**  System used, if any, such as GRADE | N/A |

| **6. Presentation of results** | |
| --- | --- |
| **Additional material**  Summary tables, flowcharts, etc, to be included in the final paper | - Protocol - Prisma Checklist - Flow chart of literature search - Table with eligibility criteria - Summary tables for results - Forest plots for meta-analyses & tables for Mann Whitney U tests - Tables for study/review limitations |
| **Outputs from review**  Papers and target journals, conference presentations, reports, etc | - The target journal for this work is PLOS One. |

| **7. Timeline for review – when do you aim to complete each stage of the review** | |
| --- | --- |
| **Protocol** | 2 weeks |
| **Literature searching** | 3 weeks |
| **Quality appraisal** | 1 week |
| **Data extraction** | 1 week |
| **Synthesis** | 3 weeks |
| **Writing up** | 4-8 weeks |
