# Supplementary figures and images for: Health-related quality of life in paediatric patients with Type 1 diabetes mellitus using insulin infusion systems. A systematic review and meta-analysis
Source: PLoS One. 2019 Jun 25;14(6):e0217655. doi: 10.1371/journal.pone.0217655 (PMC6592525; doi:10.1371/journal.pone.0217655)

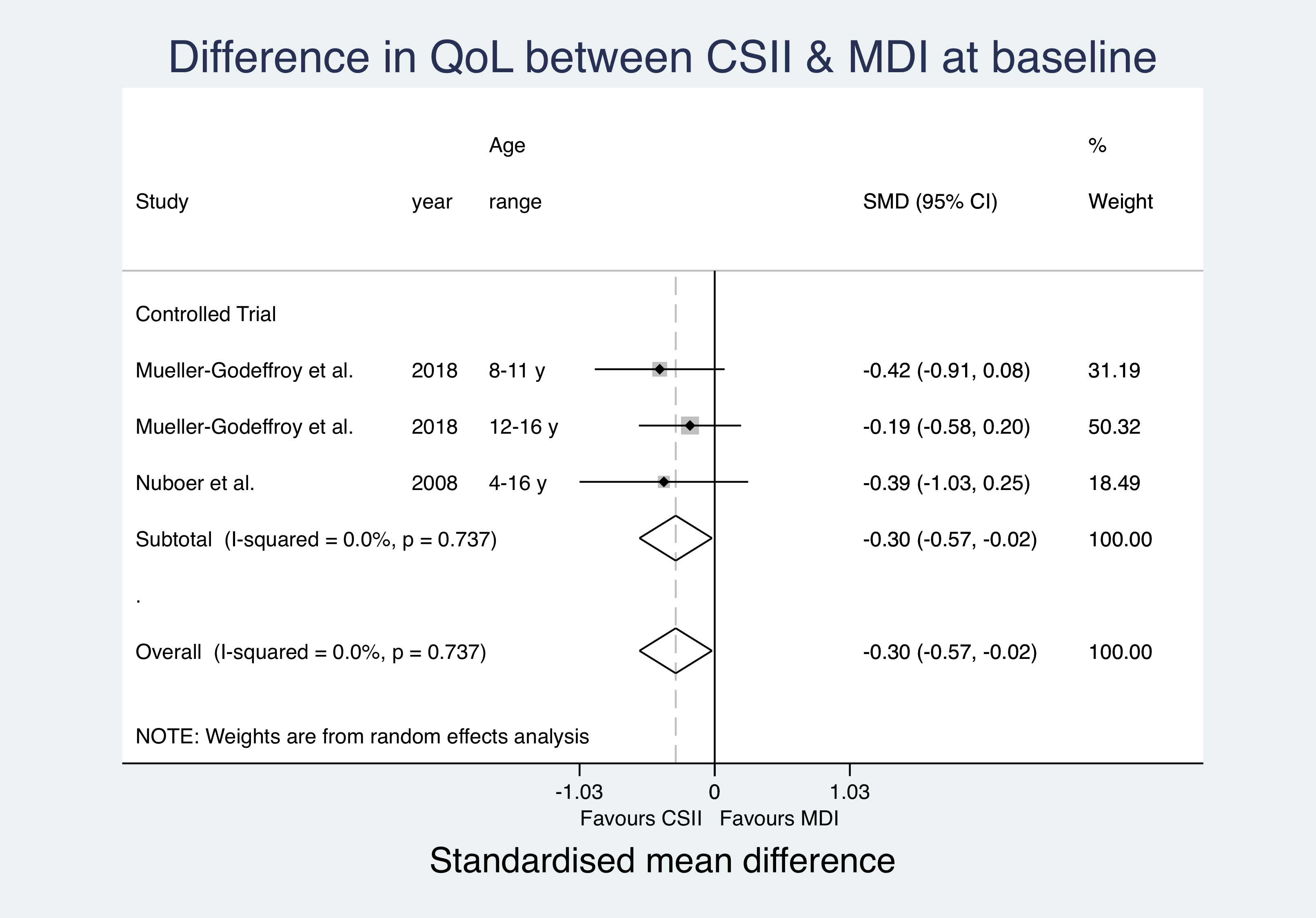

Supplement: S1 Fig — Sensitivity analysis. (TIFF) [file pone.0217655.s004.tiff]

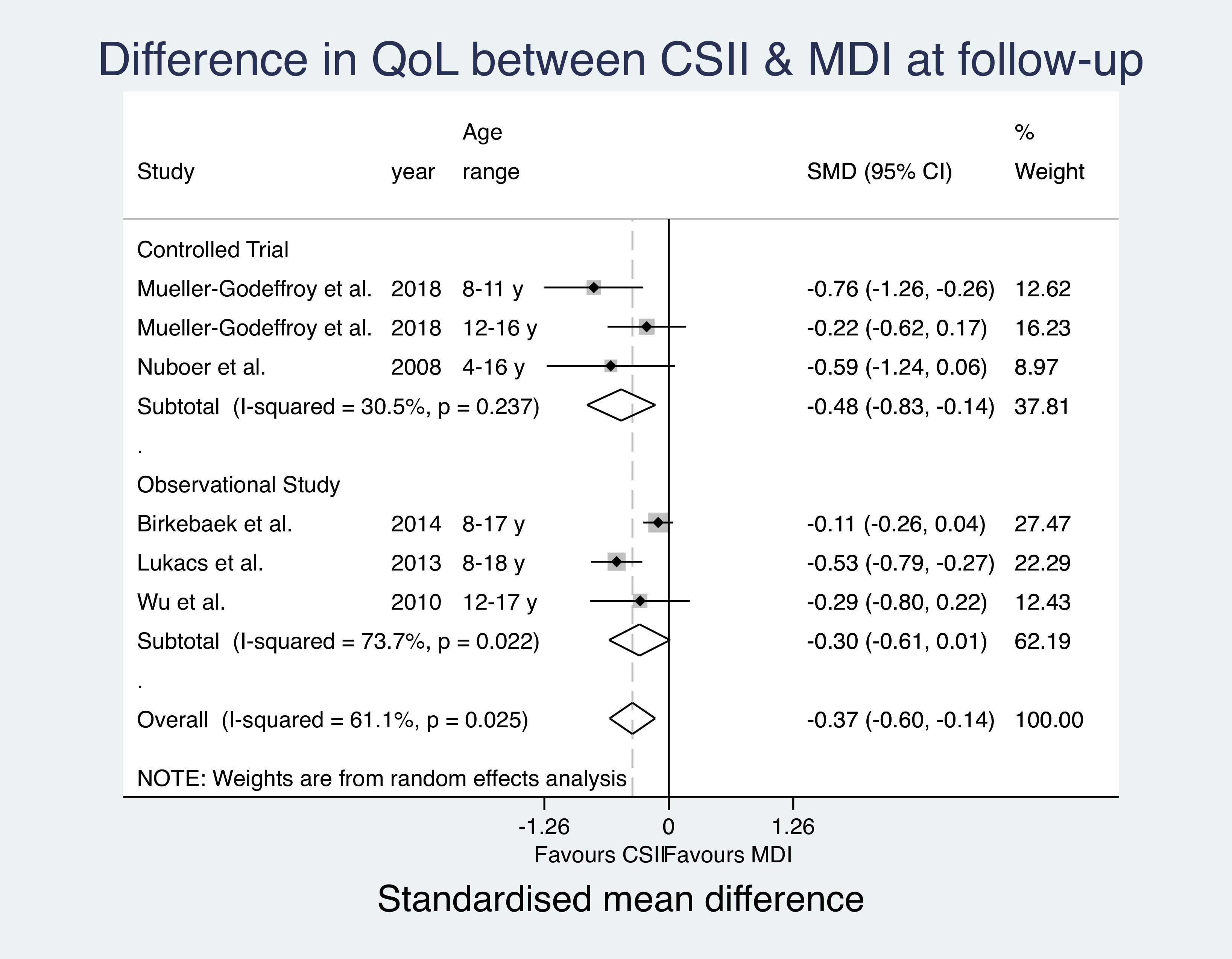

Supplement: S2 Fig — Sensitivity analysis. (TIFF) [file pone.0217655.s005.tiff]

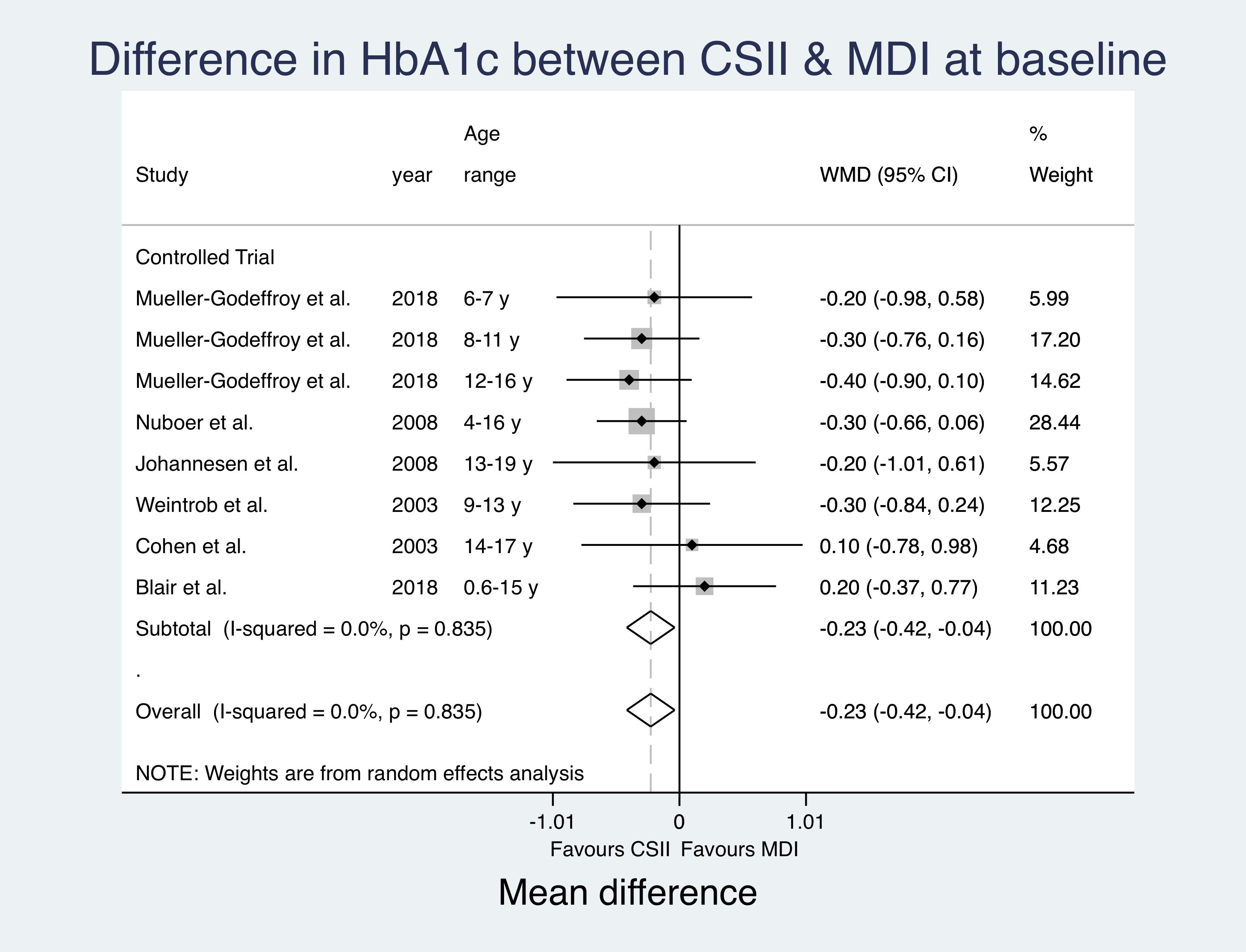

Supplement: S3 Fig — Sensitivity analysis. (TIFF) [file pone.0217655.s006.tiff]

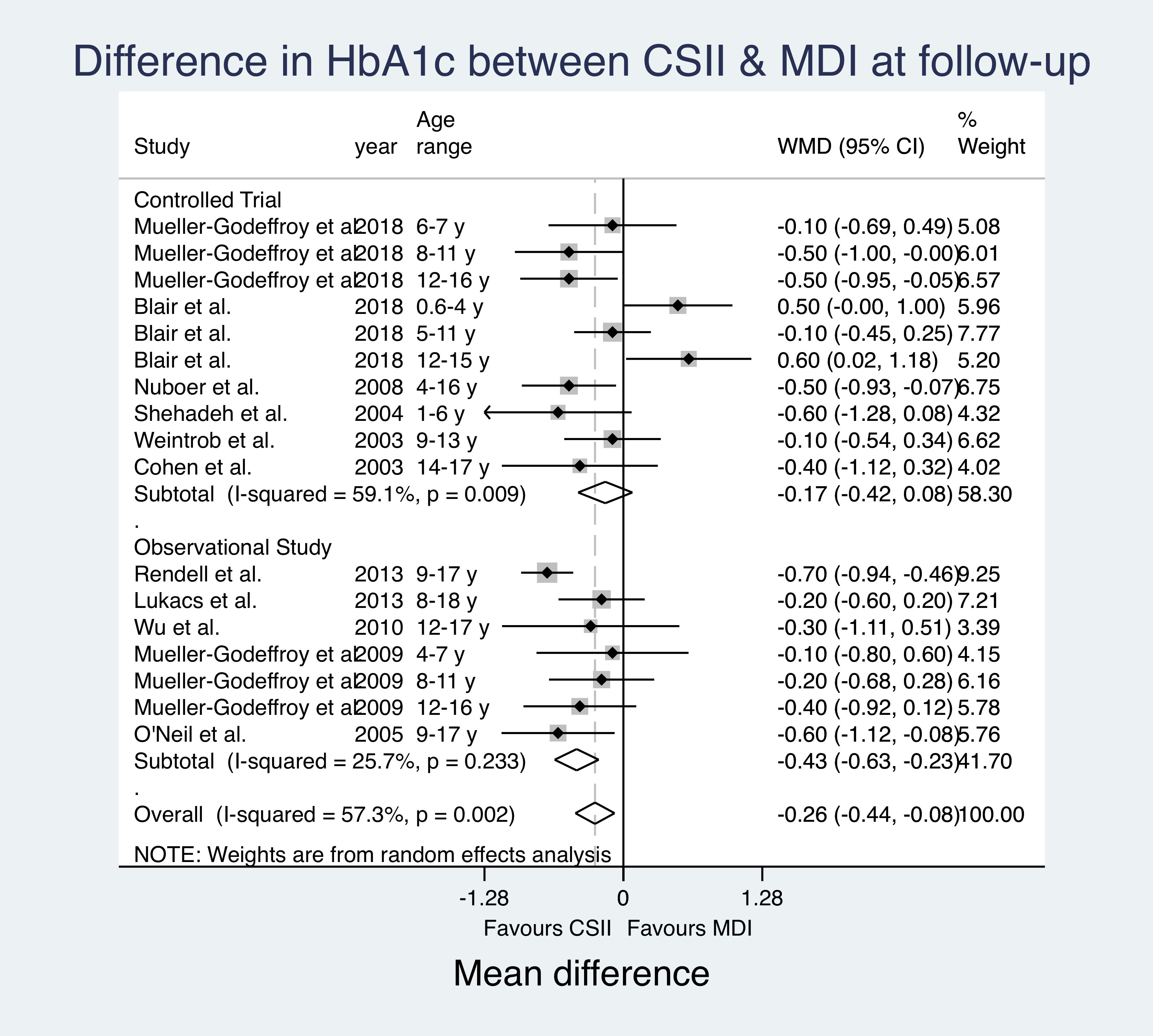

Supplement: S4 Fig — Sensitivity analysis. (TIFF) [file pone.0217655.s007.tiff]

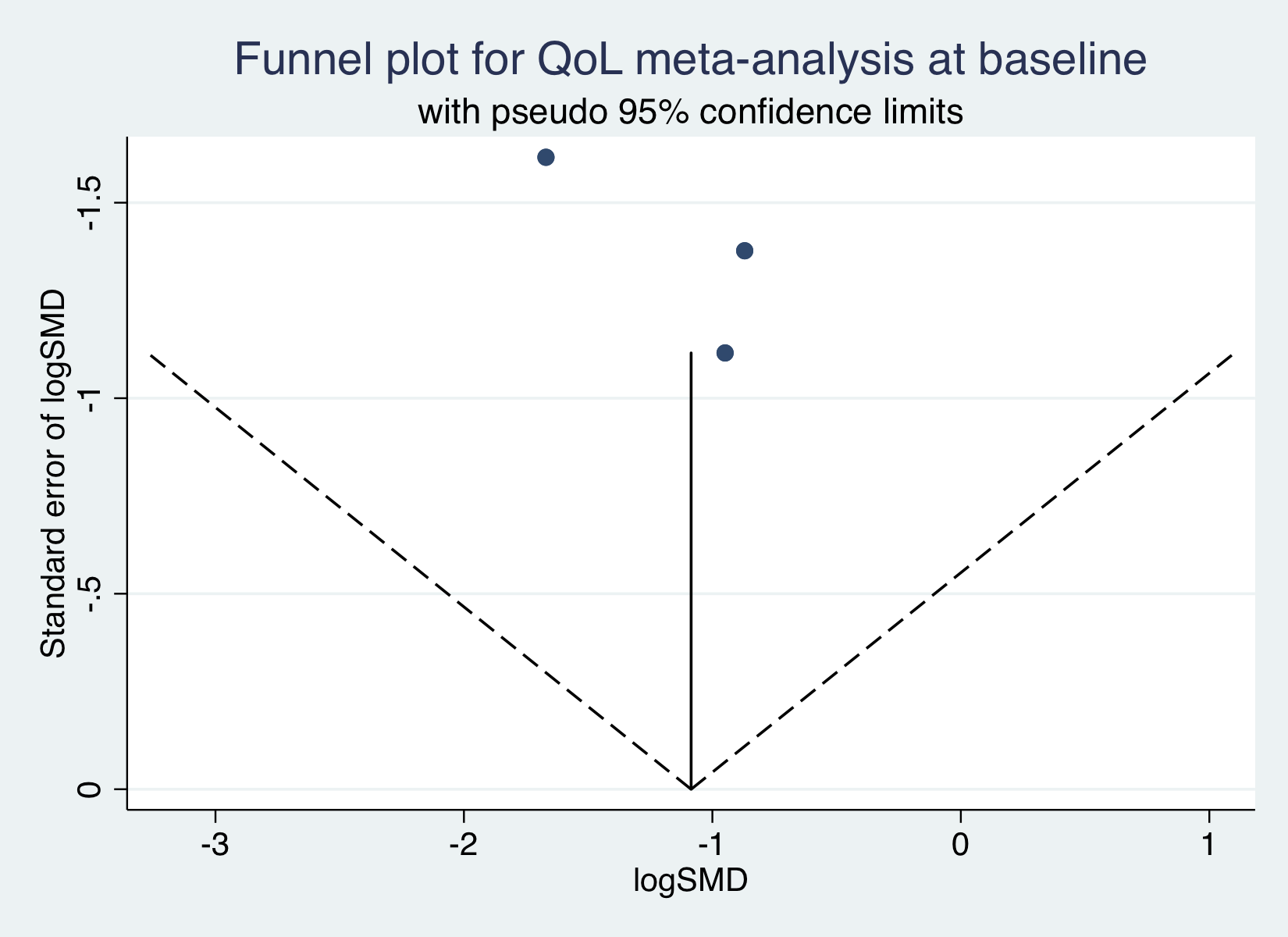

Supplement: S5 Fig — (TIFF) [file pone.0217655.s008.tiff]

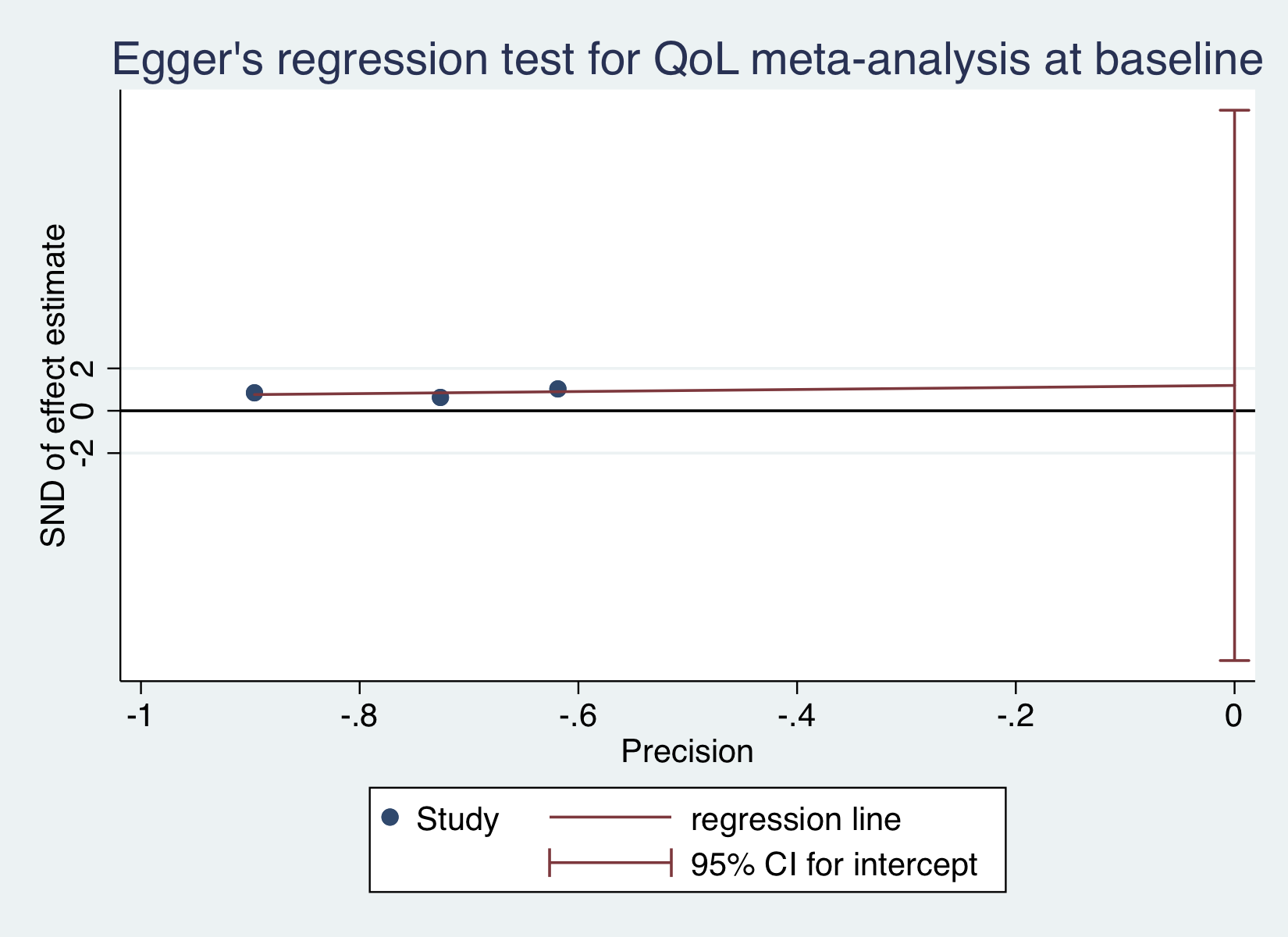

Supplement: S6 Fig — (TIFF) [file pone.0217655.s009.tiff]

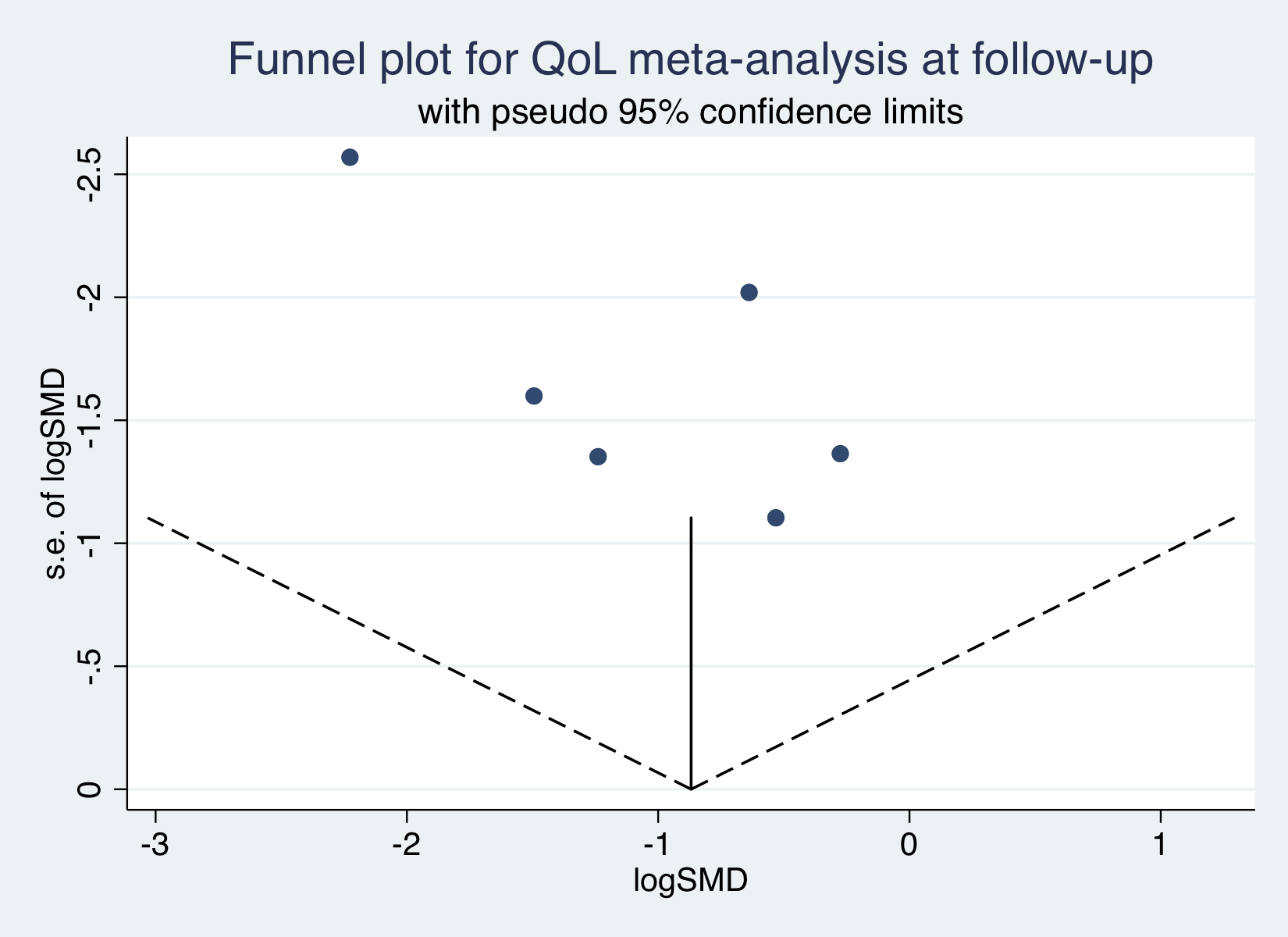

Supplement: S7 Fig — (TIFF) [file pone.0217655.s010.tiff]

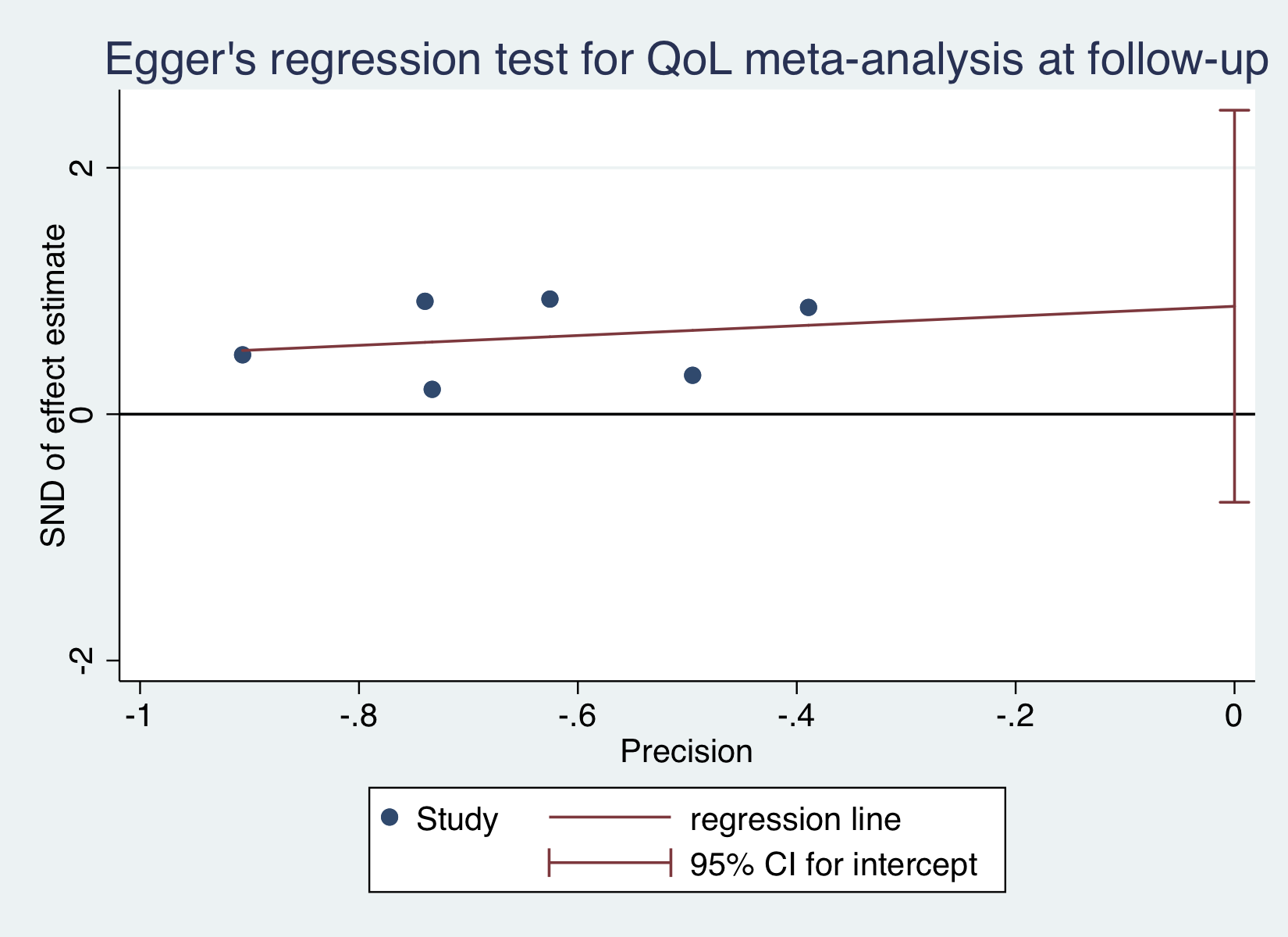

Supplement: S8 Fig — (TIFF) [file pone.0217655.s011.tiff]

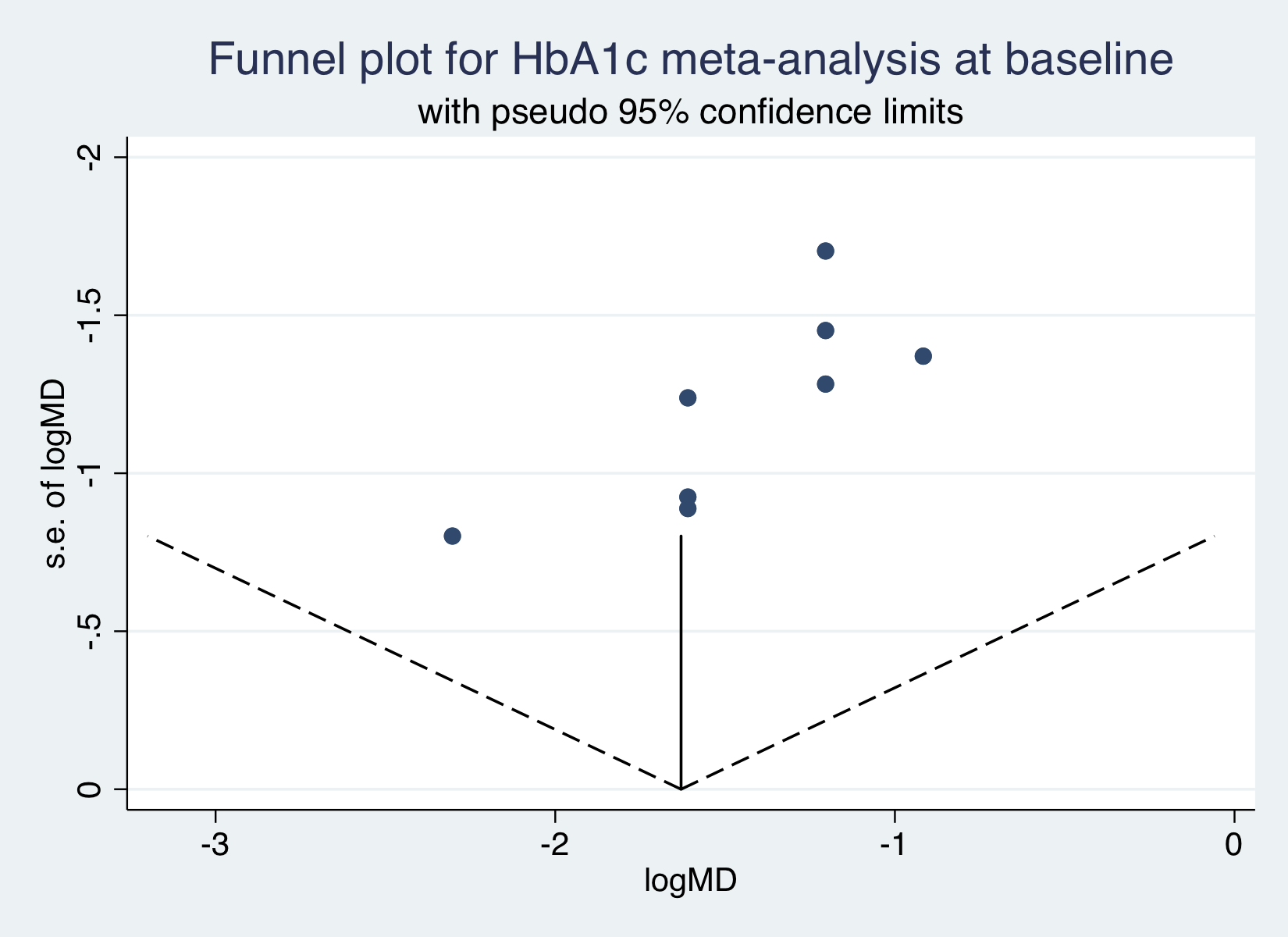

Supplement: S9 Fig — (TIFF) [file pone.0217655.s012.tiff]

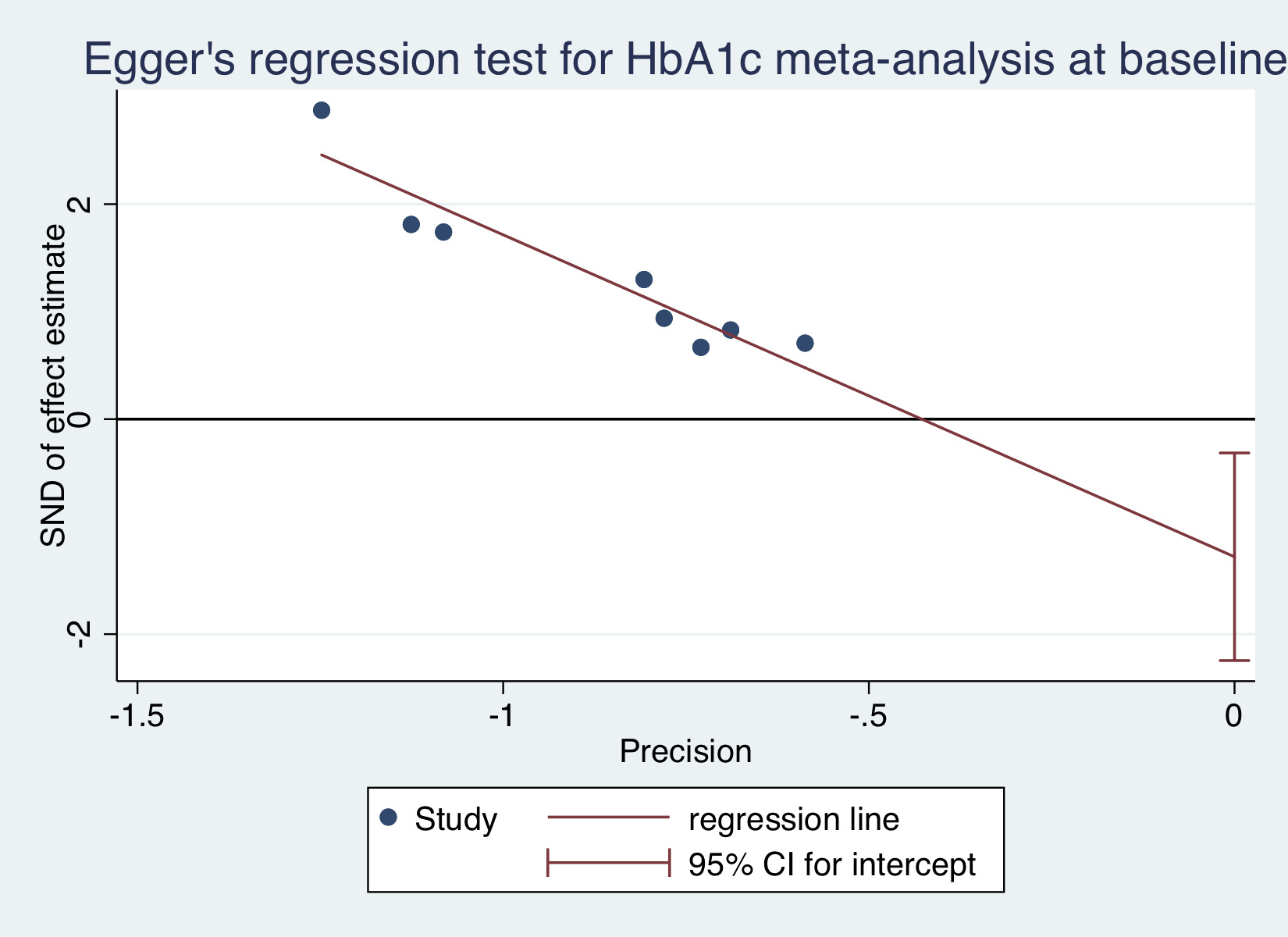

Supplement: S10 Fig — (TIFF) [file pone.0217655.s013.tiff]

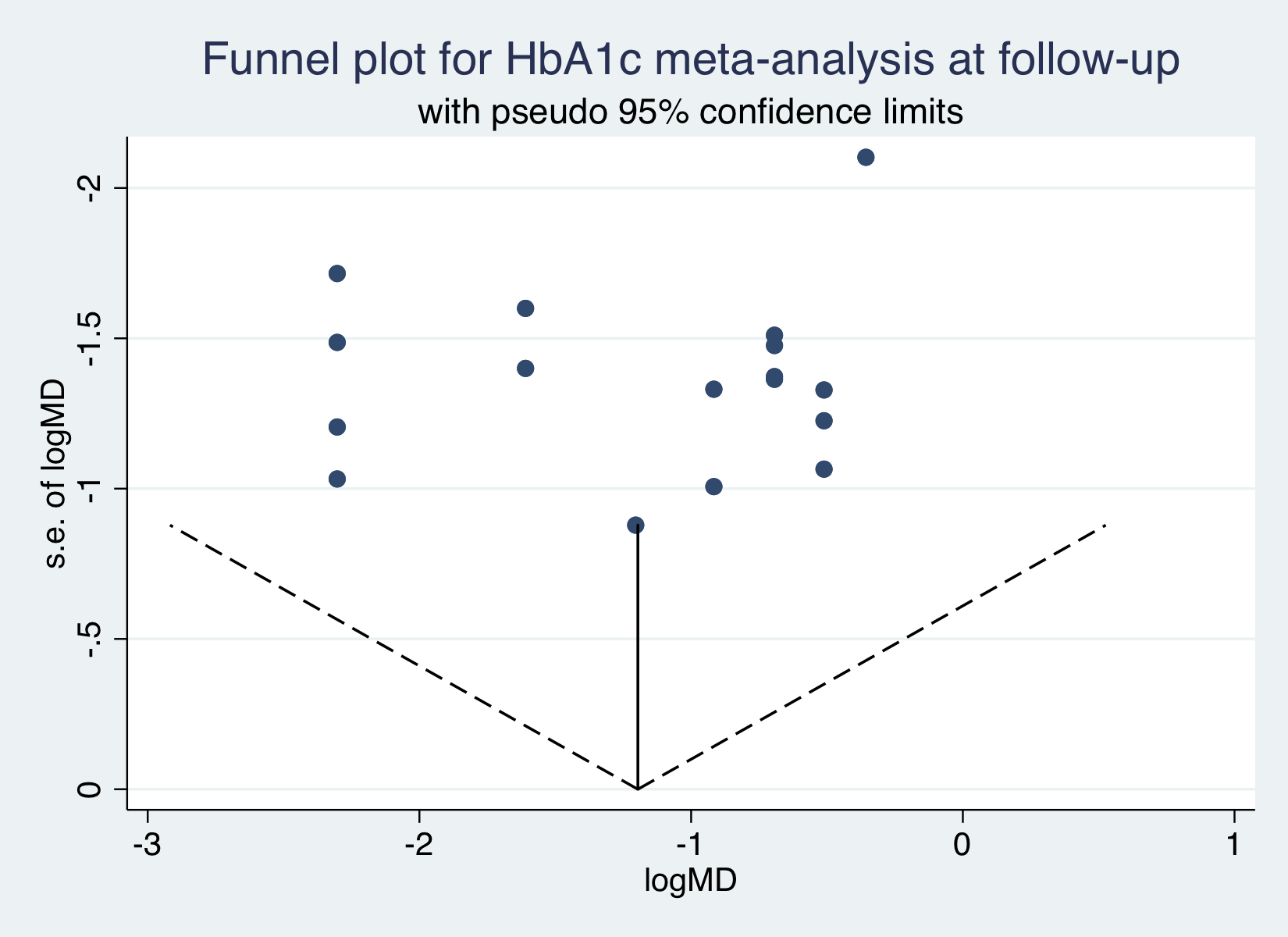

Supplement: S11 Fig — (TIFF) [file pone.0217655.s014.tiff]

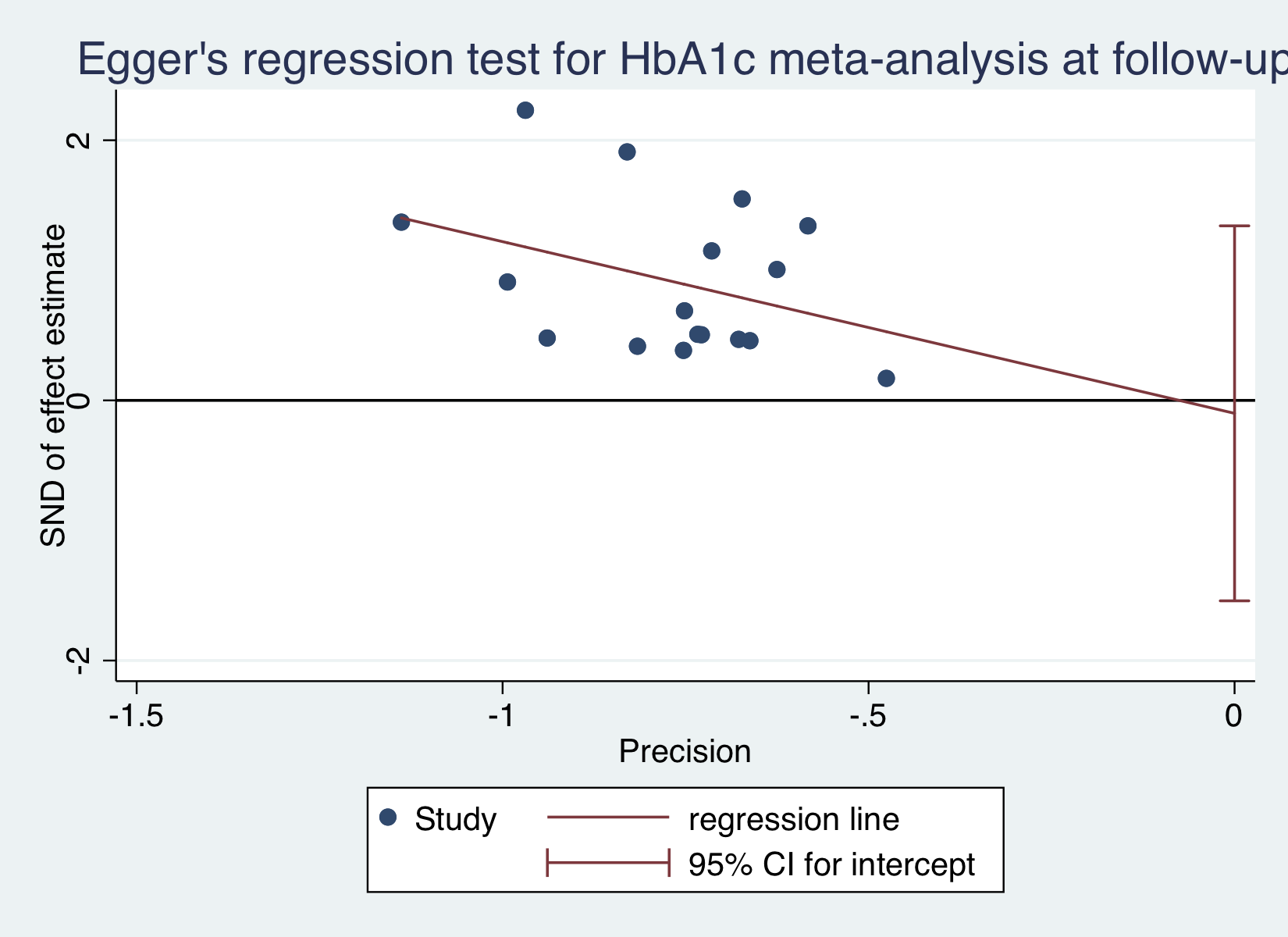

Supplement: S12 Fig — (TIFF) [file pone.0217655.s015.tiff]
